# Epigenome-wide association study in peripheral white blood cells involving insulin resistance

**DOI:** 10.1038/s41598-019-38980-2

**Published:** 2019-02-21

**Authors:** Ana Arpón, Fermín I. Milagro, Omar Ramos-Lopez, M. Luisa Mansego, José Luis Santos, José-Ignacio Riezu-Boj, J. Alfredo Martínez

**Affiliations:** 1 University of Navarra, Department of Nutrition, Food Sciences and Physiology & Centre for Nutrition Research, Pamplona, Spain; 2 Spanish Biomedical Research Centre in Physiopathology of Obesity and Nutrition (CIBERobn), Institute of Health Carlos III, Madrid, Spain; 3 Department of Nutrition, Diabetes and Metabolism, School of Medicine, Pontificia Universidad Católica de Chile, Santiago, Chile; 4 Navarra Institute for Health Research (IdiSNa), Pamplona, Spain; 5 Madrid Institute for Advanced Studies (IMDEA), IMDEA Food, Madrid, Spain

## Abstract

Insulin resistance (IR) is a hallmark of type 2 diabetes, metabolic syndrome and cardiometabolic risk. An epigenetic phenomena such as DNA methylation might be involved in the onset and development of systemic IR. The aim of this study was to explore the genetic DNA methylation levels in peripheral white blood cells with the objective of identifying epigenetic signatures associated with IR measured by the Homeostatic Model Assessment of IR (HOMA-IR) following an epigenome-wide association study approach. DNA methylation levels were assessed using Infinium Methylation Assay (Illumina), and were associated with HOMA-IR values of participants from the Methyl Epigenome Network Association (MENA) project, finding statistical associations for at least 798 CpGs. A stringent statistical analysis revealed that 478 of them showed a differential methylation pattern between individuals with HOMA-IR ≤ 3 and > 3. ROC curves of top four CpGs out of 478 allowed differentiating individuals between both groups (AUC≈0.88). This study demonstrated the association between DNA methylation in some specific CpGs and HOMA-IR values that will help to the understanding and in the development of new strategies for personalized approaches to predict and prevent IR-associated diseases.

## Introduction

Type 2 diabetes (T2D) is a worldwide major health concern and the most predominant type of diabetes^1^. According to the World Health Organisation, the global prevalence of diabetes among adults over 18 years old has risen from 4.7% in 1980 to 8.5% in 2014. Furthermore, in 2015 about 1.6 million deaths were directly attributed to diabetes^1^.

T2D is a multifactorial disease defined by the interaction of genetics and environmental factors^2^. The heritability for T2D is estimated to be between 15 and 85%. However, the genetic loci identified to date only explain 5–10% of this heritability^3^. In this context, available evidences suggest that epigenetics may be contributing to variations in gene expression and the risk for this metabolic disease^4^. In fact, recent investigations have associated the onset and progression of diabetes with specific changes in the epigenome^3,5^.

Insulin resistance (IR) is a pathological condition in which cells fail to respond properly to insulin^6^. IR is one of the most important precursors of T2D and other adversely associated cardiometabolic conditions, such as obesity, hypertension, cardiovascular disease (CVD)^7^, and metabolic syndrome^8^. IR is specifically associated with a low-grade inflammation, as well as with chronic enhancement of oxidative stress, triggering endothelial dysfunction and promoting atherogenesis^4^. Furthermore, both genetic and epigenetic factors are involved in the development of systemic IR^9^. The validated method Homeostatic Model Assessment of IR (HOMA-IR) is usually employed for measuring IR and β-cell function^10^.

Epigenetic marks are heritable changes that cannot be explained through variations in DNA nucleotide sequence^11^. These modifications are potentially reversible and can be altered by environmental factors^2^, resulting in alterations of gene expression and providing an interactive connection among genetics, specific diseases and the environment^12^.

Among the different epigenetic modifications, DNA methylation has been widely searched^13^. Some epigenome-wide association studies (EWASs) have revealed significant associations between DNA methylation and glucose homeostasis^5,14–20^, but only four of them studied some relationships with IR in different populations and approaches^5,14,15,18^. Therefore, the aim of the current work was to explore DNA methylation levels in peripheral white blood cells (PWBCs) by using an EWAS strategy with the objective of identifying epigenetic signatures associated with HOMA-IR and specifically identifying potential biomarkers that allow the discrimination of potentially hazardous HOMA-IR levels.

The assessment of epigenetic phenomena may help to understand the basis of metabolic pathway regulation, as well as the relationships between genomics and the environment influence, to promote new strategies to better understand human health and to develop novel biomarker panels related to T2D, obesity and accompanying comorbidities^20,21^.

## Results

### Participant characteristics

Anthropometric and biochemical characteristics of the participants are reported (Table 1).

**Table 1:** Anthropometric, clinical and biochemical characteristics of the study population and by project/consortium.

Variables	TOTAL		ADULTS (n = 474)*																			
			DiOGenes-UNAV		OBEPALIP		Food4Me-UNAV		GEDYMET		ICTUS		NUGENOB-UNAV		PREDIMED-UNAV		RESMENA		NormoP		OBEKIT	
	n	Values	n	Values	n	Values	n	Values	n	Values	n	Values	n	Values	n	Values	n	Values	n	Values	n	Values
Sex (females)	474	303 (64)	52	27 (52)	29	29 (100)	39	21 (54)	57	57 (100)	7	5 (71)	22	14 (64)	116	59 (51)	44	22 (50)	12	6 (50)	96	63 (66)
Age (years)	474	47.0 (14.3)	52	42.7 (5.8)	29	37.4 (7.3)	39	41.7 (10.0)	57	27.0 (6.2)	7	57.1 (7.4)	22	34.7 (9.7)	116	65.0 (3.7)	44	48.6 (10.1)	12	39.4 (5.6)	96	46.8 (9.6)
Weight (kg)	474	81.7 (19.1)	52	95.3 (17.7)	29	83.1 (9.5)	39	74.4 (14.6)	57	60.7 (8.8)	7	121.9 (15.2)	22	87.3 (20.8)	116	71.7 (9.2)	44	103.0 (18.1)	12	65.8 (9.3)	96	89.2 (13.6)
BMI (kg/m^2^)	474	30.0 (5.7)	52	33.9 (3.8)	29	31.6 (3.1)	39	26.0 (5.3)	57	24.1 (3.5)	7	44.3 (4.0)	22	31.1 (8.2)	116	27.7 (2.3)	44	36.5 (3.7)	12	22.8 (1.5)	96	31.9 (3.7)
Glucose (mg/dL)	443	102.3 (29.8)	37	99.0 (12.1)	29	89.9 (5.9)	39	91.8 (10.3)	57	78.1 (5.7)	7	120.6 (29.5)	12	102.3 (23.4)	110	121.5 (42.5)	44	122.2 (33.6)	12	85.1 (7.3)	96	95.8 (11.9)
Insulin (μUI/mL)	332	9.7 (7.0)	37	13.0 (7.1)	29	6.3 (3.3)	39	6.0 (4.6)	57	8.3 (2.7)	7	23.0 (12.2)	11	11.3 (6.4)	0	NA	44	15.8 (9.7)	12	3.6 (2.2)	96	8.5 (5.3)
HOMA-IR	332	2.4 (2.3)	37	3.2 (2.0)	29	1.4 (0.7)	39	1.4 (1.0)	57	1.6 (0.6)	7	7.1 (4.5)	11	3.0 (2.3)	0	NA	44	4.9 (3.4)	12	0.8 (0.5)	96	2.1 (1.5)
HOMA-IR > 3	78	5.7 (2.7)	19	4.7 (1.7)	1	3.3 (NA)	1	6.2 (NA)	1	3.2 (NA)	7	7.1 (4.5)	3	6.1 (2.1)	0	NA	28	6.7 (3.1)	0	NA	18	4.6 (1.5)
HOMA-IR ≤ 3	254	1.5 (0.7)	18	1.7 (0.7)	28	1.3 (0.6)	38	1.2 (0.7)	56	1.6 (0.5)	0	NA	8	1.9 (0.9)	0	NA	16	1.7 (0.4)	12	0.8 (0.5)	78	1.5 (0.7)

Values are Mean (SD), except for Sex, which is represented as number of cases (%).

*474 individuals obtained after processing the methylation raw data of 523 initial samples.

BMI: Body mass index; HOMA-IR: Homeostatic model assessment of Insulin resistance; NA: not applicable.

### DNA methylation was significantly associated with HOMA-IR

**Figure 1:**
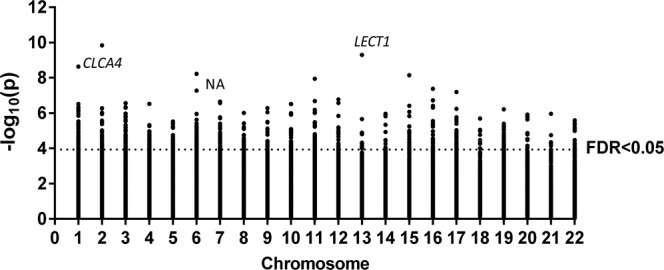
Manhattan plot of HOMA-IR-associated CpGs selected by a slope ≥|0.1|. Points above the dot line showed a False Discovery Rate (FDR) < 0.05. The three CpGs selected by slope ≥|0.1|, FDR < 0.05, Spearman’s rho, and by multiple linear regressions adjusting by sex, age, study and body mass index are marked.

**Figure 2:**
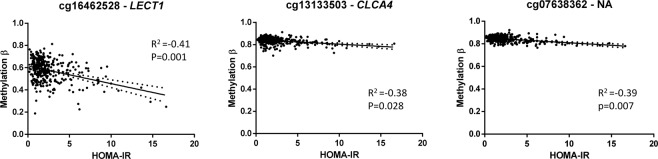
Linear regression graphs between HOMA-IR and methylation β values of the significant three CpGs selected by slope ≥|0.1|, False Discovery Rate (FDR) <0.05, and Spearman’s rho. Adjusted by study, sex, age and body mass index. Dot lines on both sides of the solid line (linear regression for correlation) represent 95% confidence band.

Methylation values of CpGs were analysed for Linear Models for Microarray Data (LIMMA) regression with HOMA-IR in 332 subjects. Significant CpGs were selected by a False Discovery Rate (FDR) cut-off of 0.05 and a slope ≥ |0.1|, obtaining 798 CpGs (Supplementary Material Table S1). The top 10 CpGs were further analysed for robustness. Spearman correlations were performed, and six CpGs were selected by having higher rho coefficient. Then, multiple linear regressions were performed adjusting by sex, age, study and body mass index (BMI), remaining three of the six CpGs significant (Table 2). These CpGs were cg13133503 (corresponding gene according to Illumina CG database *CLCA4*), cg07638362 (NA), and cg16462528 (*LECT1*), which are highlighted in a Manhattan plot (Fig. 1). Linear regression graphs between methylation values and HOMA-IR for these three CpGs are also represented (Fig. 2).

**Table 2:** Significant adjusted linear regression models of the top CpGs selected by a slope ≥ |0.1| and False Discovery Rate (FDR) < 0.05 and Spearman’s rho.

Variable (x100)^a^	β	SE	p	[95% CI]
cg16462528	−0.046	0.011	<0.001	−0.067, −0.025
cg13133503	−0.080	0.036	0.028	−0.151, −0.009
cg07638362	−0.135	0.050	0.007	−0.234, −0.037

Adjusted by study, sex, age and body mass index. CI: confidence interval; SE: standard error.

^a^β coefficients for those variables reflect increases in 0.01 units.

### Individuals with HOMA-IR > 3 showed a differential methylation pattern

Participants were classified according to the HOMA-IR cut-off of 3 in order to analyse whether methylation was differential between both groups. There were 78 individuals with HOMA-IR > 3 and 254 with HOMA-IR ≤ 3 (Table 1). Methylation values of the 798 CpGs were compared between both HOMA-IR groups. After applying the Bonferroni correction for multiple comparisons, a total of 478 CpGs showed statistically significant differences (Supplementary Material Table S2).

**Figure 3:**
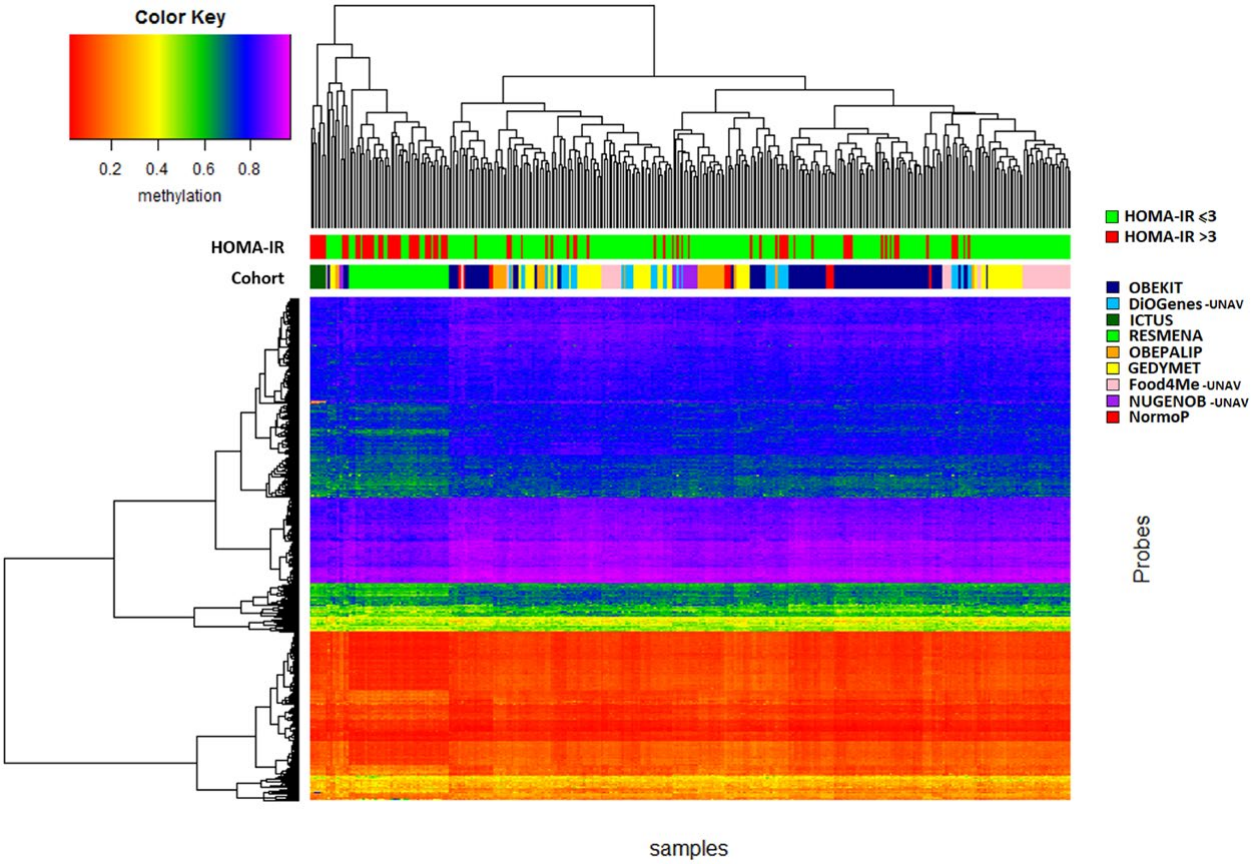
Heat map of 478 CpGs selected by Student’s *t*-test between HOMA-IR ≤3 and >3 (p < 6.26·10^−5^ after Bonferroni correction).

The resulting 478 CpGs were clustered in a heat map according to methylation patterns (Fig. 3). Two main clusters of 61 and 271 individuals were generated. The first cluster contained 62.3% of individuals with HOMA-IR > 3. However, the second cluster only included 14.8% of HOMA-IR > 3. The difference in HOMA-IR proportions of the clusters was statistically significant (p < 0.001).

### Differentially methylated CpGs between HOMA-IR groups were related to glucose and insulin pathways

**Figure 4:**
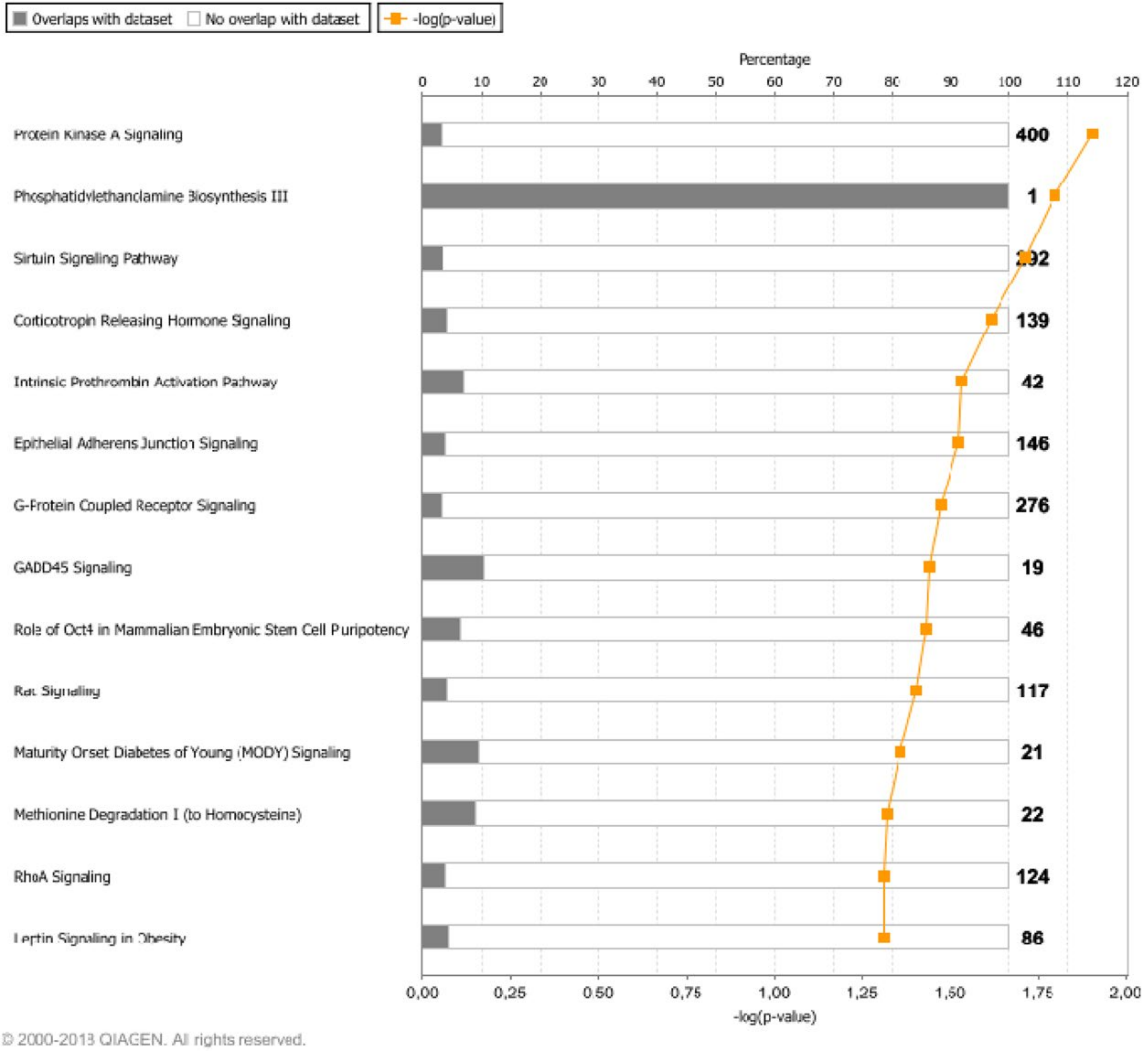
Canonical pathways from Ingenuity Pathway Analysis of 478 CpGs selected by Student’s *t*-test between HOMA-IR ≤3 and >3 (p < 6.26·10^−5^ after Bonferroni correction).

**Figure 5:**
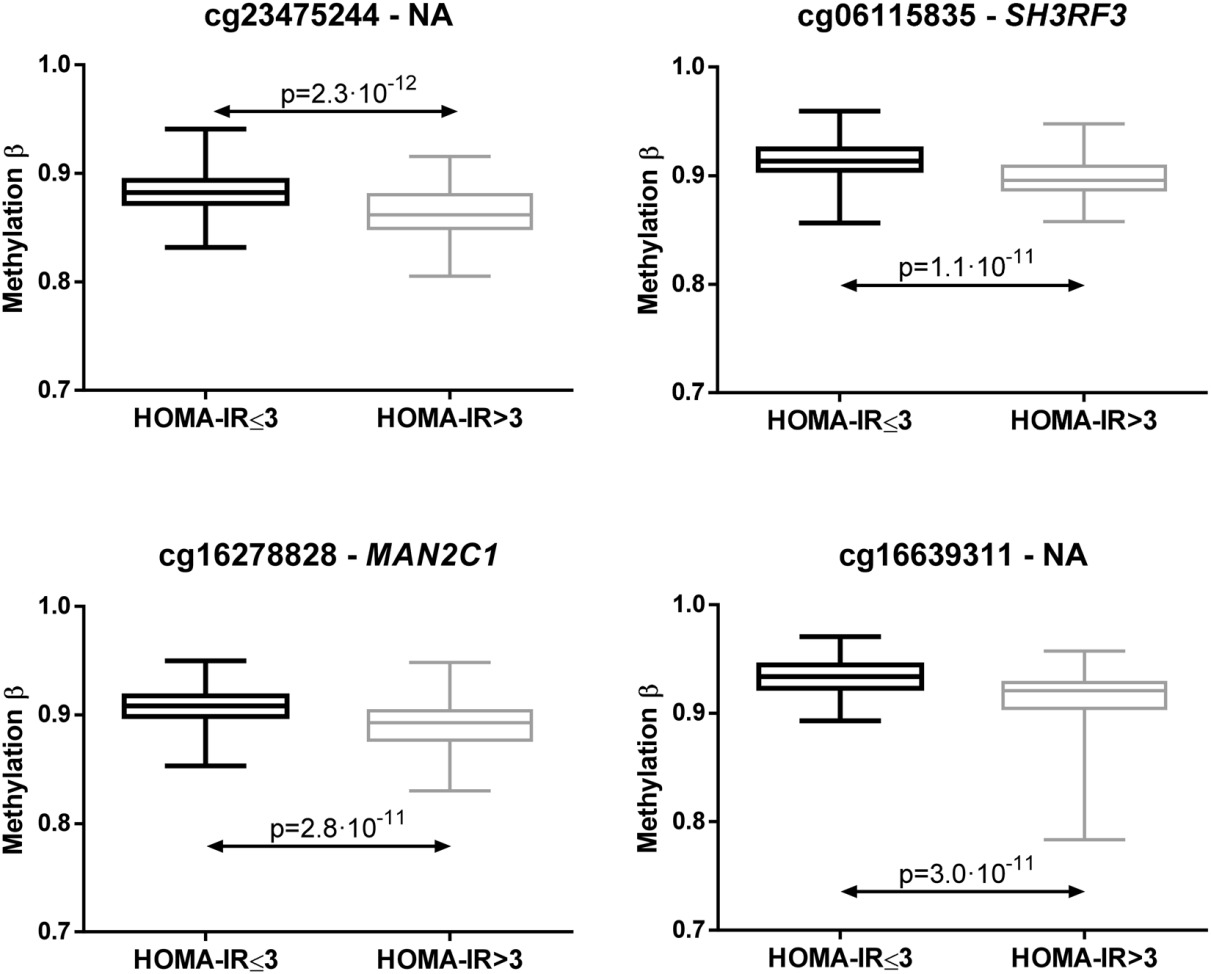
Box plots of top four CpGs selected by Student’s *t*-test between HOMA-IR ≤3 and >3 (p < 6.26·10^−5^ after Bonferroni correction). Whiskers represent minimum and maximum values.

Canonical pathways were obtained from Ingenuity Pathway Analysis (IPA) for these 478 CpGs (Fig. 4). Some of the statistically significant pathways were related to insulin and glucose, such as *Protein Kinase A Signalling*, *Sirtuin Signalling Pathway*, *G-Protein Coupled Receptor Signalling*, *Rac Signalling*, *Mature Onset Diabetes of Young (MODY) Signalling*, *RhoA Signalling*, and *Leptin Signalling in Obesity*. The top four CpGs differentially methylated between HOMA-IR ≤3 and >3 were cg23475244 (NA), cg06115835 (*SH3RF3*), cg16278828 (*MAN2C1*) and cg16639311 (NA) as illustrated (Fig. 5).

### The top four CpGs allow to differentiate between HOMA-IR ≤3 and >3

**Figure 6:**
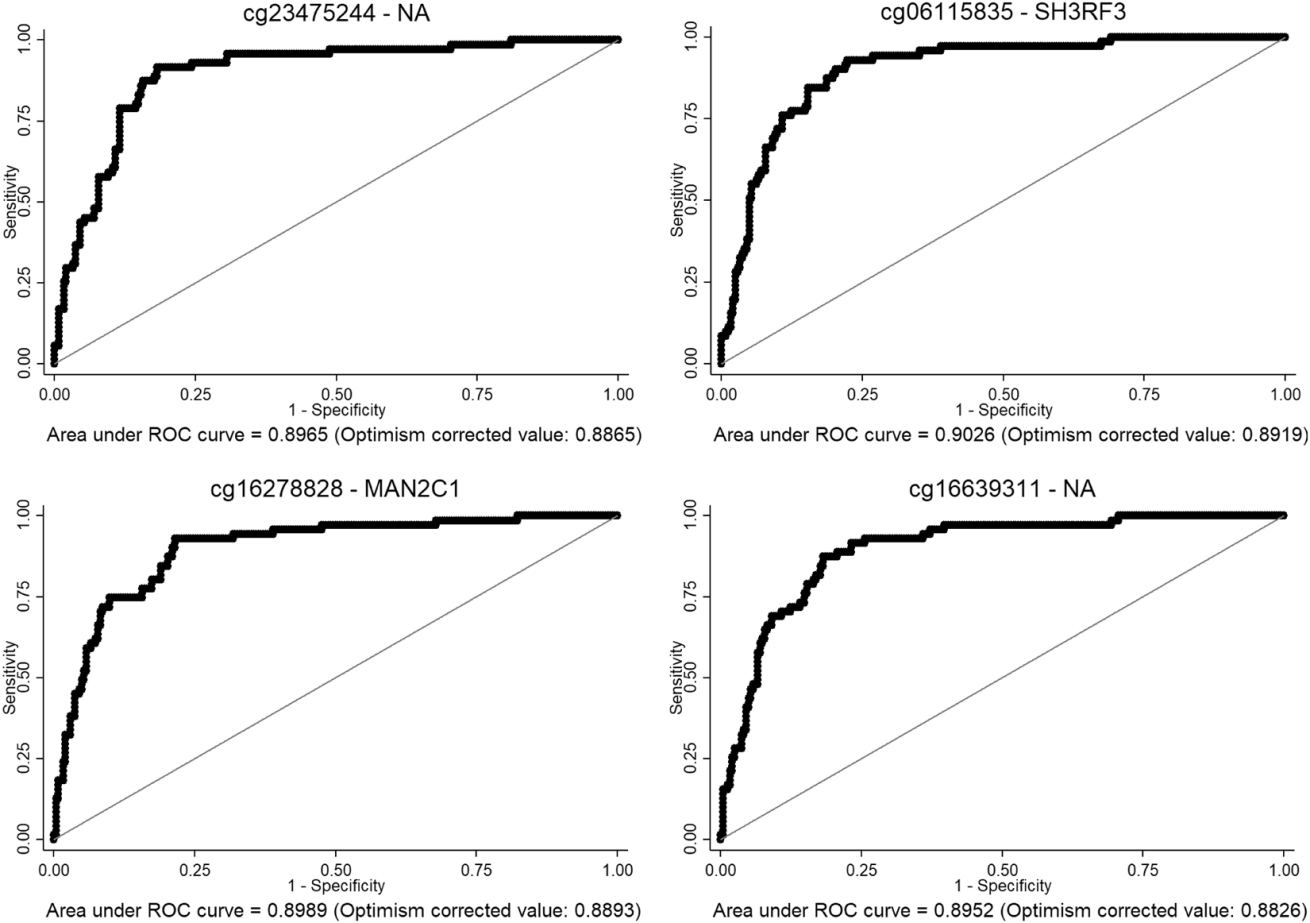
ROC curves of the top four CpGs (cg23475244, cg06115835, cg16278828, and cg16639311). Optimism corrected value was calculated using the Tibshirani’s enhanced bootstrap method described by Harrell^64^.

In order to further analyse whether methylation could differentiate between both HOMA-IR groups, the Receiver Operating Characteristic (ROC) curves adjusted by study, sex, age and BMI for the top four CpGs (cg23475244, cg06115835, cg16278828, and cg16639311) were calculated. The areas under the curve (AUC) of these CpGs were around 0.90 (AUC cg23475244 = 0.8965, AUC cg06115835 = 0.9026, AUC cg16278828 = 0.8989, and AUC cg16639311 = 0.8952), and after an internal validation (Fig. 6), the values were around 0.88 (AUC cg23475244 = 0.8865, AUC cg06115835 = 0.8919, AUC cg16278828 = 0.8893, and AUC cg16639311 = 0.8826).

## Discussion

This study involving the Methyl Epigenome Network Association (MENA) project demonstrated the association between DNA methylation in specific CpGs and HOMA-IR values. Our results also provided evidence of a differential methylation pattern between individuals with a HOMA-IR ≤3 and >3. Additionally, these data have led to the identification of four CpGs that allow us to differentiate individuals between HOMA-IR ≤ 3 and >3 with an approximate AUC of 0.88. This assay adds further insights and knowledge about the relationship between T2D-related traits and epigenetic DNA modifications.

As aforementioned, IR is a hallmark of several diseases and unhealthy cardiometabolic conditions such as T2D, CVD, hypertension, obesity^7^ and metabolic syndrome^8^. Epigenetic mechanisms have been involved in the onset and development of IR^9^. Indeed, several studies have related methylation of specific genes with HOMA-IR^3,7,8,22–31^. Nevertheless, few EWAS have been performed to date^5,14,15,18^. In line with these studies, this EWAS of the MENA project showed an association of 798 CpGs with HOMA-IR (slope ≥ |0.1| and FDR < 0.05). In our study, from the top 10 CpGs, selected ones with better association and significant after linear regressions adjusting by study, age, sex, and BMI were cg07638362 (according to Illumina CG database this CpG was not associated to any gene), cg13133503 (*CLCA4*) and cg16462528 (*LECT1*). These CpGs, to our knowledge, have not been previously described in other EWAS. However, some of the mentioned genes have been found in the list of one study. Specifically, differentially methylated regions of *LECT1* and *CLCA4* have been significantly different between diabetics and non-diabetics^32^. Both *CLCA4* and *LECT1* have been related to methylation regulation^33–35^. *CLCA4* has been involved in the activation of cAMP-dependent protein kinase A [www.genecards.org]. This pathway is intimately connected to glucose homeostasis^36^. On the other hand, *LECT1* plays a role as antiangiogenic factor in cardiac valves, preventing valvular heart diseases^37^. Methylation of this gene may be associating IR with CVD. Thus, the association of several CpGs between DNA methylation and IR detected in our study adds further support for a potential role of abnormal DNA methylation in IR^7^.

Since IR is a key feature of T2D, obesity and metabolic syndrome^7,8^, it is interesting to analyse other EWAS and methylation studies related to these adverse metabolic conditions. These investigations have been performed in several tissues such as pancreatic islets, liver, adipose tissue, skeletal muscle and blood cells^38^. There are five genes in our list that were previously related to insulin resistance (*CXCR1*, *HDAC4*, *IGFR1*, *LEPR*, and *ABCG1*)^4,5,18^. On the other hand, T2D and glycaemic traits have been associated with the following genes found in our selection *NR4A3*^32^, *KCNQ1*^39^, *IRS1*^39,40^, *SREBF1*^14,16,17,20^, *SOCS3*^14,16,17,20^, *ZNF518B*^8^, *SAMD12*^15,19^, *LY6G6E*^16^, *PHGDH*^20^, and *ABCG1*^5,14–16,18,41^. Additionally, *IRS1*^40^, *SREBF1*^18,20,^^42^, *ABCG1*^17,20,43–45^, *SOCS3*^17,44,46^, *LY6G6E*^43^ and *PHGDH*^45,47^ have also been found in EWAS analysing BMI or obesity traits. Other genes from our list that are related to obesity or BMI were *AOC3*^48^, *c7orf50*^43^, *NOD2*^20,42^, and *SLC1A5*^42^. Regarding genes associated with age, *ZNF423*^49^ and *THRB*^50^ were found in our list. In the case of smoking-associated genes, *ECE1*, *ATP8B2*, *c7orf50*, *IGF1R*, *RPL23A*, *SFRS1*^51^, *RPTOR*, *RARA*^52^, *c6orf48*^53^, and *IER3*^54^ appeared in the selection. Interestingly, the specific CpGs described for *ABCG1* (cg06500161)^5,14–18,20,42–45^, *SREBF1* (cg11024682)^14,16–18,20,42,43^, *SOCS3* (cg18181703)^14,16,17,20,46^, and *PHGDH* (cg14476101)^20,42,45,47^ were also found in our list. These four mentioned CpGs probably represent the widest described ones in relationship with T2D, obesity and other metabolic impairments in several studies with different tissues such as skeletal muscle, liver, pancreas and blood cells. Our investigation adds some new CpGs and genes to the previously described list, contributing to the knowledge and the management of IR-associated diseases.

As a novelty, our results have shown that individuals with HOMA-IR ≤ 3 or >3 exhibited a differential methylation pattern for at least 478 CpGs. Furthermore, the clustering showed that 62.3% of individuals in the first cluster had a HOMA-IR > 3. Thus, more than half of the people with similar methylation patterns presented a HOMA-IR > 3. However, the distribution of some cohorts was not heterogeneous. This situation is due to the specific recruitment requirements for each cohort. Indeed, cohorts such as RESMENA, where all the patients had metabolic syndrome, is completely found in the first cluster.

Furthermore, these 478 CpGs corresponded to some genes involved in glucose and insulin-related pathways according to IPA. For example, *Protein Kinase A Signalling*, where protein kinase A activation triggers insulin secretion in β-cells^55^; *Sirtuin Signalling Pathway*, where sirtuins influence many steps of glucose metabolism in liver, pancreas, muscle and adipose tissue^56^; and *G-Protein Coupled Receptor Signalling*, where insulin and glucagon secretion is affected by factors binding to G-protein coupled receptors on the surface of β- and α-cells^57^. Other pathways were *Rac Signalling*, which is involved in the regulation of insulin-stimulated glucose uptake^58^; *RhoA Signalling*, pathway that has been implicated in the pathogenesis of diabetes^59^; and *Leptin Signalling in Obesity*, since leptin is a regulator of glycaemic control^60^. Furthermore, *Maturity Onset Diabetes of Young (MODY) Signalling* represents the pathway of another type of diabetes that accounts for less than 2% of all diabetic cases. MODY is a monogenic form of diabetes characterized by an early onset, autosomal dominant mode of inheritance and a primary defect in pancreatic β-cell function^61^.

Only two of the top four CpGs with statistically significant differences between HOMA-IR ≤3 and >3 individuals presented associated genes according to Illumina CG database. Those genes were *SH3RF3* and *MAN2C1*. The function of *SH3RF3* is not well known, whereas *MAN2C1* is related to glycosaminoglycan (GAG) metabolism. The GAGs are heteropolysaccharides formed by a chain of repeating disaccharide units^62^. Changes in GAGs structure and function have been reported in the kidney, liver, arteries and retinal vessels of diabetics^63^.

Since methylation patterns of the 478 CpGs were able to cluster HOMA-IR individuals, we analysed the ability of the top four CpGs to differentiate between HOMA-IR ≤3 and >3 individuals. These top four CpGs distinguished HOMA-IR groups with a valuable AUC around 0.88 after an internal validation based on the optimistic correction model described by Harrell^64^, suggesting these CpGs as potential valuable biomarkers of IR.

This study was not devoid of limitations. Firstly, methylation is tissue-specific and the ideal tissue for this study would have been the pancreatic β-cells or cells from recognized insulin sensitive tissues such as skeletal muscle or white adipose tissue^65^. However, peripheral blood is the best non-invasive alternative tissue that reflects multiple metabolic and inflammatory pathways^66^, and relevant studies have demonstrated that epigenetic reprogramming may serve as a surrogate marker for metabolic disorders^41^. Interestingly, gene methylation parallelisms between peripheral blood cells and pancreatic islets have been recently reported, suggesting that blood may be used as a marker for islet DNA methylation^67^. Secondly, type I and type II error cannot be discarded, although multiple comparison tests and statistical adjustments for potential confounding factors such as sex, age, cohorts, DNA methylation chips, and cell composition heterogeneity have been performed. Thirdly, a validation sample would have been useful to corroborate the results in the selected genes. Unfortunately, this sample was not available. However, in order to resolve this issue and correct the overestimation of AUC, an internal validation using a bootstrap method^64^ was performed, obtaining similar results. Further studies are needed to verify the relationship between the selected CpGs and HOMA-IR. Finally, due to the cross-sectional feature of the study, methylation cannot be defined as a cause or consequence of cardiometabolic conditions. Remarkably, although there is an epigenetic programming during the first stages of human development^68^, Wahl *et al*. have described methylation alterations as a cause of higher BMI and adiposity^20^.

Epigenetic gene regulation, and specifically, DNA methylation, is playing a role in the pathogenesis of many complex disorders, including T2D, obesity or metabolic syndrome^22^. There is great interest to perform methylation profiling in peripheral blood to find potential methylation disease-related associations and use specific DNA methylated regions as biomarkers^69^. In summary, this study found associations between DNA methylation and IR, a hallmark of T2D, with a differential methylation pattern between individuals with HOMA-IR ≤ 3 and > 3 in genes that are mainly involved in glucose and insulin-related pathways, and suggested four CpGs as biomarkers of IR. These results will hopefully contribute to the understanding of some epigenetic mechanisms that may regulate glycaemic traits, such as HOMA-IR, and the risk of T2D, as well as provide the basis for creating personalized strategies to predict, prevent and treat IR-associated diseases.

## Subjects and Methods

### Participants

The MENA project was conducted in 523 adult participants from available cohorts at the University of Navarra (UNAV): DiOGenes-UNAV with n = 58^70^, OBEPALIP with n = 29^71^, Food4Me-UNAV with n = 42^72^, GEDYMET with n = 57^73^, ICTUS with n = 7^74^, NUGENOB-UNAV with n = 42^75^, PREDIMED-UNAV with n = 129^76,77^, RESMENA with n = 47^78^, OBEKIT with n = 100^79^ and NormoP with n = 12. However, only 474 final samples were available after the data processing explained in detail below.

Study designs, characteristics, inclusion and exclusion criteria were described for each study cohort, except for NormoP, whose design has not yet been described. All of them were approved by the Research Ethics Committee of the University of Navarra (CEI-UN, Pamplona, Spain), except for GEDYMET, which was approved by the Ethics committee of the School of Medicine, Pontificia Universidad Católica de Chile (Santiago, Chile), in compliance with the Helsinki Declaration of ethical principles for medical research involving human subjects. All participants provided written informed consent.

The NormoP cohort participants recruitment started in 2016 in the University of Navarra (Pamplona, Spain). Eligible participants were self-declared healthy individuals, >18 years old, and had a BMI of between 18.5 and 24.9 kg/m^2^. Exclusion criteria included pregnancy, type I diabetes, severe renal and digestive diseases, hydroelectrolitic disorders, acute CVD, cardiac arrhythmias, ictus, neoplasia, anaemia, eating disorders, pharmacological treatment, and dietary supplements that may affect the results.

### Study variables

Anthropometric measurements and the metabolic profile were obtained from databases of the aforementioned cohorts, which followed validated protocols. Data of some characteristics were not available for all the 474 participants. IR was estimated using the validated HOMA-IR index method^10^.

### DNA extraction and DNA methylation analysis

Venous blood samples were drawn on EDTA tubes. Genomic DNA was extracted from PWBCs using the MasterPure^TM^ DNA Purification kit (Epicenter, Madison, WI), whose quality was assessed with the Pico Green dsDNA Quantitation Reagent (Invitrogen, Carlsbad, CA). High-quality DNA samples (500 ng) were treated with bisulfite using the EZ-96 DNA Methylation Kit (Zymo Research Corporation, Irvine, CA) according to the manufacturer’s instructions, converting cytosine into uracil. DNA methylation levels were measured by microarray with the Infinium Human Methylation 450 K bead chip technology (Illumina, San Diego, CA, USA) in all the cohorts, except OBEKIT, which was performed with Infinium MethylationEPIC beadchip (Illumina). This analysis was conducted in the Unidad de Genotipado y Diagnóstico Genético from Fundación Investigación Clínico de Valencia, as detailed elsewhere^80^.

### Treatment of methylation raw data

Beta-values have been used as metrics to measure methylation levels. Beta-value in methylation experiments is the estimate of the methylation level using the ratio of the methylation probe intensity and the overall intensity, corresponding to the percentage of methylation on a specific site^81^. After obtaining intensity data using ChAMP package for R v.1.11.0^82^ as described elsewhere^83^, the filtering process was performed in probes with a detection p-value above 0.01 in one or more samples, probes with a beadcount <3 in at least 5% of samples, non-CpG probes, probes with SNPs^84^, probes that align to multiple locations^84^ and probes located on the X or Y chromosomes.

From the 523 initial participants, samples with a failed CpG fraction above 0.01 were eliminated (n = 20), leaving 503 individuals. After filtering probes, intra-cell type normalization was done using Subset-quantile Within Array Normalization (SWAN) method to avoid the bias introduced by the Infinium type 2 probe design^85^. In order to assess the similarity of normalized methylation samples in both batches and the pooled data, multidimensional scaling plots based on top of 1000 most variable probes were performed. A total of 29 samples failed to fulfil this requisite, which left 474 participants for the subsequent analyses.

After SWAN normalization, magnitude of batch effects were assessed and corrected using the ComBat normalization method, which is an empirical Bayes based method to correct for technical variation related to the slide^86,87^. Furthermore, differences in methylation resulting from differences in cellular heterogeneity were corrected using the Houseman procedure^88^.

The data discussed in this publication have been deposited in NCBI’s Gene Expression Omnibus^89^ and are accessible through GEO Series accession number GSE115278 (https://www.ncbi.nlm.nih.gov/geo/query/acc.cgi?acc=GSE115278).

### Statistical analysis

After pre-processing, LIMMA package from the R statistical software^82^ was used to compute a linear regression between DNA methylation values and HOMA-IR. A total of 332 subjects from the MENA project showed data for both variables (Table 1). This analysis was adjusted by the effect of confounding factors such as sex, age, study and bead chip. Raw p-values were corrected using the Benjamini-Hochberg procedure for multiple comparisons, and a FDR cut-off of 0.05 and a slope ≥ |0.1| were used as statistically significant thresholds. The top 10 CpGs were analysed for robustness with Spearman correlations and then, linear regressions between HOMA-IR and methylation adjusted for study, sex, age, and BMI were also performed for the six selected CpGs.

The cut-off for HOMA-IR differs for different races, ages, genders, diseases, complications, etc.^90^ and no reference value has been established^91^. Since there is no consensus for the HOMA-IR cut point and in order to facilitate the analysis of this metabolically heterogeneous group, a cut-off of HOMA-IR = 3 was chosen, corresponding to a value between the 75th and 80th percentiles, which are established as cut points by International Diabetes Federation (IDF) and Adult Treatment Panel III (ATPIII) for metabolic syndrome^92^. No influences in terms of races were considered, since more than 92% of the individuals were Caucasian in the MENA project and additionally, the study has been considered as a covariate in the analyses. Moreover, some studies have previously used this cut-off for HOMA-IR^93,94^. Differentially methylated CpGs between individuals with HOMA-IR > 3 and HOMA-IR ≤ 3 were explored using two-tailed Student’s t-test with Bonferroni correction. A p-value < 6.26·10^−5^ was considered significant. Adjusted (for study, sex, age, and BMI) ROC curves were performed to determine the AUC of the top selected CpGs distinguishing individuals between HOMA-IR ≤ 3 or > 3. Furthermore, an internal validation using a correction for optimistic prediction was performed according to Tibshirani’s enhanced bootstrap method described by Harrell^64^ in order to evaluate the overestimation of the model.

Statistical calculations were performed with STATA version 12.0 (Stata Corp, College Station, TX, USA), unless otherwise indicated. Manhattan plots, correlation graphs and box plots were produced using GraphPad Prism 6 (Graph-Pad Software, CA, USA). The heat map was created with the R software^82^ using library gplots and the heatmap.2 function.

### Ingenuity Pathway Analysis

Differentially methylated CpGs between individuals with HOMA-IR > 3 and HOMA-IR ≤ 3 were analysed by IPA software (Qiagen Redwood City, CA, USA, www.ingenuity.com) as defined in the package. Predefined pathways and functional categories of the Ingenuity Knowledge Base were used in order to detect associated pathways and relevant gene regulatory networks^95^. Pathway analyses were performed with IPA’s Core Analysis module. Canonical pathways with a p < 0.05 after Fisher’s test were defined as a statistically significant overrepresentation of input genes in a given process.

## Supplementary information

Supplementary material

## Data Availability

The data have been deposited in NCBI’s Gene Expression Omnibus^89^ and are accessible through GEO Series accession number GSE115278 (https://www.ncbi.nlm.nih.gov/geo/query/acc.cgi?acc=GSE115278).
