# Supplementary material for: Epigenome-wide association study in peripheral white blood cells involving insulin resistance
Source: Sci Rep. 2019 Feb 21;9:2445. doi: 10.1038/s41598-019-38980-2 (PMC6385280; doi:10.1038/s41598-019-38980-2)
Supplement: Supplementary file 1 — Supplementary material [file 41598_2019_38980_MOESM1_ESM.pdf]

Epigenome-wide association study in peripheral white blood cells involving insulin resistance

Ana Arpón, Fermín I. Milagro, Omar Ramos-Lopez, M. Luisa Mansego, José Luis Santos, José-Ignacio Riezu-Boj, J. Alfredo Martínez

Page 2 → Table S1: Selection of 798 CpGs after linear regression by FDR<0.05 and slope ≥ |0.1|.

Page 33 → Table S2: Selection of 478 CpGs after comparing individuals with HOMA≤3 and >3 (Student's t-test + Bonferroni correction).

Table S1: Selection of 798 CpGs after linear regression by FDR<0.05 and slope ≥ |0.1|.

CpG	UCSC_RefGene_Accession	UCSC_RefGene_Group	UCSC_CpG_Islands_Name	p	FDR
cg22051399	NA	NA	NA	1.44E-10	4.90E-05
cg16462528	LECT1;LECT1	NM_001011705;NM_007015	Body;Body	4.96E-10	8.46E-05
cg13133503	CLCA4;CLCA4	NR_024602;NM_012128	Body;Body	2.31E-09	0.00026276
cg13108601	FRS3;PRICKLE4	NM_006653;NM_013397	TSS1500;TSS1500	5.88E-09	0.00048823
cg02613818	IGF1R	NM_000875	Body	7.16E-09	0.00048823
cg19749898	HCCA2	NM_053005	Body	1.14E-08	0.00064765
cg02793828	NA	NA	NA	4.27E-08	0.00207921
cg07638362	NA	NA	NA	5.32E-08	0.00226558
cg14315232	NA	NA	NA	6.47E-08	0.00245104
cg04559178	NA	NA	NA	1.72E-07	0.00556053
cg10293354	NA	NA	NA	1.89E-07	0.00556053
cg05935374	RAB30	NM_014488	TSS200	2.08E-07	0.00556053
cg20703375	ARPC1A	NM_006409	Body	2.24E-07	0.00556053
cg19260567	CNTNAP2	NM_014141	Body	2.72E-07	0.00556053
cg03806828	RAB21	NM_014999	TSS200	2.72E-07	0.00556053
cg09242721	TRNT1	NM_182916	TSS200	2.74E-07	0.00556053
cg22964775	LPHN3	NM_015236	TSS1500	3.02E-07	0.00556053
cg07908574	SLC45A1	NM_001080397	Body	3.07E-07	0.00556053
cg04238871	FAM53B	NM_014661	5'UTR	3.10E-07	0.00556053
cg04737885	ADCY9	NM_001116	3'UTR	3.88E-07	0.00636839
cg15924985	NA	NA	NA	3.92E-07	0.00636839
cg04724873	KIAA1751	NM_001080484	Body	4.66E-07	0.00650242
cg11390741	ITGA9	NM_002207	Body	4.69E-07	0.00650242
cg06180061	C16orf91	NM_001010878	Body	4.90E-07	0.00650242
cg15236063	SELK	NM_021237	3'UTR	5.04E-07	0.00650242

cg01809437	KLHL36	NM_024731	Body	5.12E-07	0.00650242
cg08052428	RALGDS;RALGDS	NM_001042368;NM_006266	Body;1stExon	5.29E-07	0.00650242
cg19391498	GALM	NM_138801	TSS1500	5.34E-07	0.00650242
cg01841050	KRT34	NM_021013	TSS1500	5.77E-07	0.00672909
cg02902865	DHRS3	NM_004753	TSS1500	5.92E-07	0.00672909
cg02048674	RPL13AP5;RPL13A	NR_026712;NM_012423	Body;Body	6.15E-07	0.00676325
cg22636056	NA	NA	NA	6.58E-07	0.00700489
cg00459550	NA	NA	NA	7.62E-07	0.00786681
cg16278828	MAN2C1	NM_006715	Body	8.51E-07	0.00804755
cg21607453	NCRNA00092	NR_024129	Body	8.91E-07	0.00804755
cg05422174	KIRREL3;KIRREL3	NM_001161707;NM_032531	Body;Body	9.20E-07	0.00804755
cg20853880	KLF11	NM_003597	Body	9.48E-07	0.00804755
cg00931944	NA	NA	NA	9.74E-07	0.00804755
cg22870994	NA	NA	NA	9.76E-07	0.00804755
cg18543035	NA	NA	NA	1.02E-06	0.00804755
cg10400362	AJAP1;AJAP1	NM_018836;NM_001042478	Body;Body	1.04E-06	0.00804755
cg24511898	ATG4B;ATG4B;THAP4	NM_178326;NM_013325;NM_015963	TSS1500;TSS1500;TSS200	1.06E-06	0.00804755
cg23162967	CBLB	NM_170662	TSS200	1.07E-06	0.00804755
cg06665941	IFNAR2;IFNAR2;IFNAR2	NM_207585;NM_207584;NM_000874	5'UTR;5'UTR;5'UTR	1.09E-06	0.00804755
cg20087917	C14orf133;AHSA1	NM_022067;NM_012111	TSS200;TSS1500	1.09E-06	0.00804755
cg16104446	MCM4;MCM4;PRKDC;PRKDC	NM_182746;NM_005914;NM_001081640;NM_006904	Body;Body;TSS1500;TSS1500	1.11E-06	0.00804755
cg19450145	TULP4;TULP4	NM_020245;NM_001007466	Body;Body	1.11E-06	0.00804755
cg09984469	CYP26B1	NM_019885	Body	1.13E-06	0.00804755
cg23104823	PRPF39;PRPF39	NM_017922;NM_017922	1stExon;5'UTR	1.20E-06	0.00831494
cg20261243	NCOA5	NM_020967	TSS200	1.23E-06	0.00831494
cg11688874	WAC;WAC;WAC;WAC;WAC	NM_100486;NM_016628;NM_016628;NM_100486;NR_024557	1stExon;5'UTR;1stExon;5'UTR;Body	1.24E-06	0.00831494

cg10525720	NA	NA	NA	1.43E-06	0.0091724
cg00472528	NA	NA	NA	1.44E-06	0.0091724
cg16091269	NA	NA	NA	1.47E-06	0.0091724
cg09392827	NA	NA	NA	1.48E-06	0.0091724
cg23205818	RGS6	NM_004296	Body	1.52E-06	0.00923743
cg02737268	CDC25B;CDC25B;CDC25B	NM_021873;NM_004358;NM_021872	Body;Body;Body	1.61E-06	0.00963584
cg03160445	LNP1;TOMM70A;LNP1	NM_001085451;NM_014820;NM_001085451	5'UTR;TSS200;1stExon	1.73E-06	0.00996398
cg17484877	PTPRN2;PTPRN2;PTPRN2	NM_002847;NM_130842;NM_130843	Body;Body;Body	1.74E-06	0.00996398
cg08723637	NA	NA	NA	1.75E-06	0.00996398
cg13947929	CRHR1;CRHR1;CRHR1;CRHR1	NM_001145148;NM_004382;NM_001145147;NM_001145146	Body;Body;Body;Body	1.89E-06	0.01056913
cg15877295	UBN2	NM_173569	TSS1500	1.96E-06	0.01078959
cg11814422	SNORD58A;RPL17;SNORD58B;RPL17	NR_002571;NM_000985;NR_002572;NM_001035006	TSS1500;5'UTR;TSS200;5'UTR	2.05E-06	0.01109319
cg22811384	NA	NA	NA	2.21E-06	0.01178206
cg05744073	MIR132	NR_029674	Body	2.26E-06	0.01181472
cg25188760	NA	NA	NA	2.29E-06	0.01181472
cg03227783	HLA-DPB2	NR_001435	Body	2.42E-06	0.01224402
cg13713052	FAM162A;CCDC58	NM_014367;NM_001017928	TSS1500;TSS200	2.44E-06	0.01224402
cg24962827	THRB;THRB;THRB	NM_000461;NM_001128176;NM_001128177	Body;Body;Body	2.53E-06	0.01230445
cg04889221	CCDC37	NM_182628	Body	2.54E-06	0.01230445
cg14193886	LIF;LIF	NM_002309;NM_002309	5'UTR;1stExon	2.56E-06	0.01230445
cg02304481	FAM178B;FAM178B	NM_001122646;NM_001122646	5'UTR;1stExon	2.70E-06	0.01280718
cg06836020	LOC440354;LOC595101	NR_002473;NR_002453	Body;Body	2.83E-06	0.01310199
cg17876831	SLC5A10;SLC5A10	NM_001042450;NM_152351	Body;Body	2.84E-06	0.01310199
cg22219795	NA	NA	NA	2.90E-06	0.01310467
cg21304211	C1orf183;C1orf183;C1orf183	NM_198926;NM_019099;NM_019099	Body;1stExon;5'UTR	2.92E-06	0.01310467
cg09853839	NA	NA	NA	3.04E-06	0.01330323

cg19741148	NA	NA	NA	3.04E-06	0.01330323
cg19597318	NFASC;NFASC;NFASC;NFASC;NFASC	NM_015090;NM_001160333;NM_001005388;<br>NM_001005389;NM_001160332	5'UTR;5'UTR;5'UTR;5'UTR;5'UTR	3.09E-06	0.01334721
cg14156446	NR6A1;NR6A1	NM_033334;NM_001489	3'UTR;3'UTR	3.19E-06	0.01358452
cg02694576	PTDSS2	NM_030783	Body	3.34E-06	0.0140408
cg14044465	NA	NA	NA	3.40E-06	0.01413129
cg01963897	NA	NA	NA	3.48E-06	0.01414112
cg05301188	KLF11	NM_003597	Body	3.48E-06	0.01414112
cg21963643	CACNA1E	NM_000721	TSS1500	3.61E-06	0.01422584
cg22920258	MCHR1	NM_005297	TSS1500	3.62E-06	0.01422584
cg14421779	NA	NA	NA	3.65E-06	0.01422584
cg10251347	NRD1;NRD1;NRD1;NRD1	NM_002525;NM_002525;NM_001101662;NM_001101662	5'UTR;1stExon;1stExon;5'UTR	3.67E-06	0.01422584
cg22901726	NA	NA	NA	3.77E-06	0.01422584
cg16783478	GRIN2A;GRIN2A;GRIN2A	NM_001134407;NM_000833;NM_001134408	Body;Body;Body	3.83E-06	0.01422584
cg22341273	HEATR7A;HEATR7A;HEATR7A	NM_001099281;NM_001099280;NM_032450	Body;Body;Body	3.88E-06	0.01422584
cg20752695	NA	NA	NA	3.89E-06	0.01422584
cg01726103	FAM65B	NM_014722	TSS1500	3.89E-06	0.01422584
cg08250273	ST7;ST7OT4;ST7OT1;ST7	NM_018412;NR_002329;NR_002330;NM_021908	TSS200;TSS1500;Body;TSS200	3.96E-06	0.01422584
cg09332650	PRR12	NM_020719	Body	3.99E-06	0.01422584
cg14932796	COIL	NM_004645	1stExon	4.01E-06	0.01422584
cg18358845	ZNF454	NM_182594	Body	4.08E-06	0.01432482
cg13885205	NA	NA	NA	4.22E-06	0.0146651
cg22081084	LRRC8B	NM_001134476	TSS200	4.40E-06	0.0151354
cg05254095	ZDHHC7;ZDHHC7	NM_001145548;NM_017740	3'UTR;3'UTR	4.49E-06	0.01513971
cg04598128	NA	NA	NA	4.52E-06	0.01513971
cg05093429	TCL6	NR_028288	Body	4.53E-06	0.01513971
cg04948475	UGT2A3;UGT2A3	NM_024743;NR_024010	Body;Body	4.76E-06	0.01568255

cg18062721	VGLL4;VGLL4	NM_014667;NM_001128219	Body;Body	4.78E-06	0.01568255
cg19545348	RCOR3;RCOR3;RCOR3;RCOR3	NM_018254;NM_001136224;NM_001136225;NM_001136223	Body;Body;Body;Body	5.14E-06	0.0166509
cg17599958	AP2A2	NM_012305	Body	5.18E-06	0.0166509
cg17009526	FAT1	NM_005245	Body	5.31E-06	0.01685222
cg13241473	TFEB;TFEB	NM_001167827;NM_007162	5'UTR;TSS1500	5.36E-06	0.01685222
cg10355458	MBD3	NM_003926	3'UTR	5.40E-06	0.01685222
cg01353569	HBP1	NM_012257	5'UTR	5.44E-06	0.01685222
cg17745433	OBSCN;OBSCN	NM_052843;NM_001098623	Body;Body	5.51E-06	0.01692716
cg19613905	MNS1;MNS1	NM_018365;NM_018365	1stExon;5'UTR	5.58E-06	0.01697989
cg09991951	NA	NA	NA	5.73E-06	0.01712542
cg07145255	DEPDC1B;DEPDC1B	NM_018369;NM_001145208	Body;Body	5.78E-06	0.01712542
cg19951269	NA	NA	NA	5.78E-06	0.01712542
cg01223673	INSC	NM_001031853	TSS200	5.83E-06	0.01712542
cg23924911	NA	NA	NA	6.26E-06	0.01797324
cg04318006	SNTB1	NM_021021	1stExon	6.27E-06	0.01797324
cg09529537	JMY	NM_152405	TSS200	6.27E-06	0.01797324
cg21757872	BTBD11;BTBD11	NM_001017523;NM_001018072	TSS200;Body	6.40E-06	0.01801082
cg15944459	PLA2G6;BAIAP2L2;PLA2G6	NM_001004426;NM_025045;NM_003560	3'UTR;TSS1500;3'UTR	6.41E-06	0.01801082
cg16198129	ZNF662;ZNF662	NM_207404;NM_001134656	Body;Body	6.46E-06	0.01801082
cg04605980	NA	NA	NA	6.58E-06	0.01801082
cg09016822	IER3	NM_003897	TSS200	6.62E-06	0.01801082
cg12402495	C1orf43;C1orf43;UBAP2L;UBAP2L;C1orf43	NM_015449;NM_138740;NM_014847;NM_001127320;NM_001098616	1stExon;1stExon;TSS1500;5'UTR;1stExon	6.68E-06	0.01801082
cg00584840	NA	NA	NA	6.70E-06	0.01801082
cg24613691	NA	NA	NA	6.77E-06	0.01801082
cg22136672	NA	NA	NA	6.80E-06	0.01801082
cg06124975	SOBP	NM_018013	TSS1500	6.82E-06	0.01801082

cg02804275	NA	NA	NA	7.24E-06	0.01889851
cg18547667	NA	NA	NA	7.28E-06	0.01889851
cg18654971	MIR449C;CDC20B;CDC20B;CDC20B	NR_031572;NM_001170402;NM_001145734;NM_152623	TSS1500;TSS1500;TSS1500;TSS1500	7.32E-06	0.01889851
cg06936159	ZNF707;ZNF707;ZNF707;ZNF707;ZNF707;ZNF707	NM_001100599;NM_173831;NM_001100598; NM_173831;NM_001100598;NM_001100599	1stExon;1stExon;1stExon;5'UTR;5'UTR;5'UTR	7.46E-06	0.01912047
cg18624108	COL11A2;COL11A2;COL11A2	NM_080679;NM_080681;NM_080680	Body;Body;Body	7.60E-06	0.01931119
cg09015484	NA	NA	NA	7.65E-06	0.01931119
cg23436918	BZW2;ANKMY2;BZW2;BZW2	NM_001159767;NM_020319;NR_027624;NM_014038	TSS200;TSS200;TSS200;TSS200	7.72E-06	0.01934502
cg03531754	FAM83A;FAM83A	NM_032899;NM_207006	TSS1500;TSS1500	7.79E-06	0.01938837
cg10150615	LOC391322	NM_001144931	TSS200	7.99E-06	0.01946077
cg21838880	ZNF461;ZNF461	NM_153257;NM_153257	1stExon;5'UTR	8.00E-06	0.01946077
cg11580090	CYB5R1	NM_016243	3'UTR	8.05E-06	0.01946077
cg14039479	GPR26	NM_153442	3'UTR	8.05E-06	0.01946077
cg13412395	NR4A3;NR4A3;NR4A3	NM_006981;NM_173198;NM_173199	TSS1500;TSS1500;TSS1500	8.17E-06	0.01961891
cg15999833	HECW1	NM_015052	Body	8.31E-06	0.01981871
cg13507084	CNTFR;CNTFR	NM_147164;NM_001842	Body;Body	8.38E-06	0.01983649
cg03226245	SDHAF1;SDHAF1	NM_001042631;NM_001042631	1stExon;5'UTR	8.64E-06	0.02031788
cg01339558	TIPRL;TIPRL	NM_001031800;NM_152902	Body;Body	8.73E-06	0.02036028
cg00356500	TNXB	NM_019105	Body	8.78E-06	0.02036028
cg16139199	ESCO1	NM_052911	5'UTR	8.96E-06	0.02063552
cg19932227	ADPRH	NM_001125	TSS200	9.10E-06	0.02080373
cg11287903	DCLRE1A;NHLRC2;DCLRE1A	NM_014881;NM_198514;NM_014881	5'UTR;TSS1500;1stExon	9.15E-06	0.02080373
cg08470053	ZNF160;ZNF160;ZNF160	NM_033288;NM_198893;NM_001102603	5'UTR;5'UTR;5'UTR	9.40E-06	0.02112705
cg25184507	CYB5D1;LSMD1	NM_144607;NM_032356	TSS1500;Body	9.42E-06	0.02112705
cg23851043	MOBKL3;MOBKL3;MOBKL3	NM_199482;NM_001100819;NM_015387	5'UTR;Body;Body	9.56E-06	0.02130979
cg24430189	NA	NA	NA	9.70E-06	0.02147522
cg20584198	STAC3	NM_145064	Body	1.00E-05	0.02191558

cg23622695	NA	NA	NA	1.00E-05	0.02191558
cg15401862	KCNMB4	NM_014505	3'UTR	1.02E-05	0.022139
cg13116086	POM121L10P	NR_024593	TSS1500	1.03E-05	0.022139
cg19020758	NA	NA	NA	1.04E-05	0.02219217
cg09540629	KNDC1	NM_152643	Body	1.06E-05	0.02247626
cg11110248	MANBA	NM_005908	TSS200	1.07E-05	0.02247626
cg12315590	C3orf37;C3orf37	NM_001006109;NM_020187	TSS1500;TSS200	1.07E-05	0.02247626
cg04835971	RASSF3	NM_178169	3'UTR	1.08E-05	0.02250063
cg09422450	NA	NA	NA	1.08E-05	0.02251295
cg21319458	BAZ2B;BAZ2B	NM_013450;NM_013450	1stExon;5'UTR	1.10E-05	0.0225298
cg13769834	NA	NA	NA	1.10E-05	0.0225298
cg00232805	ANKRD36	NM_001164315	1stExon	1.12E-05	0.0225776
cg18701983	TNXB	NM_019105	5'UTR	1.13E-05	0.0225776
cg19306036	GREB1	NM_014668	Body	1.13E-05	0.0225776
cg22351924	IFI6;IFI6;IFI6	NM_022872;NM_002038;NM_022873	Body;Body;Body	1.13E-05	0.0225776
cg04792380	NA	NA	NA	1.14E-05	0.0225776
cg12404679	KLHL30	NM_198582	Body	1.14E-05	0.0225776
cg08458400	CNR1;CNR1;CNR1;CNR1;CNR1;CNR1	NM_001160259;NM_001160226;NM_001160258;<br>NM_001160260;NM_033181;NM_016083	Body;Body;Body;Body;Body;Body	1.15E-05	0.0225776
cg13435243	LRRC8A;LRRC8A;LRRC8A	NM_001127245;NM_001127244;NM_019594	Body;Body;Body	1.16E-05	0.0225776
cg19094530	NA	NA	NA	1.17E-05	0.0225776
cg25235380	ANKFY1;ANKFY1	NM_020740;NM_016376	TSS200;TSS200	1.17E-05	0.0225776
cg00004939	C14orf181;C14orf181	NM_207442;NM_207442	3'UTR;1stExon	1.17E-05	0.0225776
cg02326858	ZNF687	NM_020832	TSS1500	1.18E-05	0.0225776
cg00997699	PLXNA2	NM_025179	Body	1.19E-05	0.0226536
cg00031105	RXRB	NM_021976	Body	1.20E-05	0.0226536
cg00832650	FLNC;FLNC	NM_001127487;NM_001458	Body;Body	1.22E-05	0.02290126

cg21683619	LEPR;LEPR;LEPR	NM_001003679;NM_002303;NM_001003680	Body;Body;Body	1.23E-05	0.02290126
cg11327366	FAM189A1	NM_015307	Body	1.23E-05	0.02290126
cg21689228	MSX1	NM_002448	TSS1500	1.24E-05	0.02290126
cg00455514	NA	NA	NA	1.24E-05	0.02290126
cg23219925	NA	NA	NA	1.25E-05	0.02299412
cg10581632	CDK2;CDK2;CDK2;SILV;CDK2	NM_052827;NM_001798;NM_001798;NM_006928;NM_052827	5'UTR;1stExon;5'UTR;TSS1500;1stExon	1.28E-05	0.02308375
cg00410419	COMMD6;COMMD6	NM_203497;NM_203495	Body;Body	1.28E-05	0.02308375
cg08531746	ATP11A;ATP11A	NM_015205;NM_032189	Body;Body	1.28E-05	0.02308375
cg18784586	GPRC5A;GPRC5A	NM_003979;NM_003979	1stExon;5'UTR	1.29E-05	0.02308375
cg17361449	TTPAL;TTPAL	NM_001039199;NM_024331	TSS200;TSS200	1.34E-05	0.02377131
cg23182539	TIAL1;TIAL1	NM_001033925;NM_003252	Body;Body	1.34E-05	0.02377131
cg19964273	NA	NA	NA	1.35E-05	0.02377131
cg11906607	GNG7	NM_052847	5'UTR	1.36E-05	0.02377131
cg15084903	THEG;THEG	NM_016585;NM_199202	TSS200;TSS200	1.36E-05	0.02377131
cg25256723	F5	NM_000130	TSS200	1.38E-05	0.02391311
cg17794996	C6orf48;C6orf48	NM_001040438;NM_001040437	Body;Body	1.38E-05	0.02391311
cg24002010	TMEM86B;SAPS1	NM_173804;NM_014931	TSS1500;3'UTR	1.39E-05	0.02391311
cg03649497	CACNA1G;CACNA1G;CACNA1G;CACNA1G;CACNA1G;CACNA1G;CACNA1G;CACNA1G;CACNA1G;CACNA1G;CACNA1G;CACNA1G;CACNA1G;CACNA1G;CACNA1G	NM_198384;NM_198379;NM_198380;NM_198377;NM_198385;NM_018896;NM_198396;NM_198386;NM_198376;NM_198397;NM_198388;NM_198383;NM_198382;NM_198378;NM_198387	Body;Body;Body;Body;Body;Body;Body;Body;Body;Body;Body;Body;Body;Body;Body	1.41E-05	0.02422014
cg08980198	NA	NA	NA	1.42E-05	0.02422014
cg17983632	NA	NA	NA	1.44E-05	0.02448221
cg04618340	NA	NA	NA	1.46E-05	0.02464946
cg05177851	EIF2B3;EIF2B3	NM_001166588;NM_020365	5'UTR;5'UTR	1.47E-05	0.02472716
cg00170564	BTBD11	NM_001018072	Body	1.49E-05	0.0248262
cg20009378	GRK1	NM_002929	Body	1.49E-05	0.02485361
cg16275967	NDUFS2;NDUFS2	NM_004550;NM_001166159	Body;1stExon	1.51E-05	0.02489702

cg04772948	LOC284788;LOC284788	NR_027090;NR_027089	TSS200;TSS200	1.51E-05	0.02489702
cg02582774	COQ5	NM_032314	Body	1.53E-05	0.02510443
cg21850852	ITGB4;ITGB4;ITGB4	NM_001005731;NM_001005619;NM_000213	Body;Body;Body	1.54E-05	0.02518711
cg00570269	C13orf33	NM_032849	Body	1.56E-05	0.02536814
cg24769972	AP2A2	NM_012305	Body	1.58E-05	0.02536814
cg05573182	MAFB	NM_005461	TSS1500	1.58E-05	0.02536814
cg03438024	NA	NA	NA	1.59E-05	0.02536814
cg12393716	TTC9C;HNRNPUL2	NM_173810;NM_001079559	TSS1500;TSS1500	1.60E-05	0.02555559
cg13116438	STK19;STK19;DOM3Z;STK19	NM_004197;NM_032454;NM_005510;NR_026717	Body;Body;TSS1500;Body	1.62E-05	0.02555559
cg08664652	GOSR1;GOSR1;GOSR1	NM_001007024;NM_004871;NM_001007025	TSS200;TSS200;TSS200	1.62E-05	0.02555559
cg03771840	TRIM15	NM_033229	3'UTR	1.63E-05	0.02555559
cg03399598	GALNT7	NM_017423	Body	1.66E-05	0.02566003
cg07306531	ZNF75A	NM_153028	TSS200	1.66E-05	0.02566003
cg22523851	NA	NA	NA	1.66E-05	0.02566003
cg22753607	ZCCHC7	NM_032226	TSS200	1.67E-05	0.02566003
cg04517374	CABLES2	NM_031215	Body	1.69E-05	0.02566003
cg08977639	NA	NA	NA	1.69E-05	0.02566003
cg24545908	NA	NA	NA	1.69E-05	0.02566003
cg09198968	NA	NA	NA	1.72E-05	0.02566003
cg00017601	PTK2B;PTK2B;PTK2B;PTK2B	NM_173176;NM_173175;NM_173174;NM_004103	Body;Body;Body;Body	1.73E-05	0.02566003
cg20847018	NA	NA	NA	1.73E-05	0.02566003
cg15649111	CPA5;CPA5;CPA5	NM_001127441;NM_001127442;NM_080385	Body;Body;Body	1.74E-05	0.02566003
cg21473183	GNLY;GNLY	NM_006433;NM_012483	3'UTR;3'UTR	1.74E-05	0.02566003
cg15772133	LTBP1;LTBP1;LTBP1;LTBP1;LTBP1	NM_000627;NM_206943;NM_001166265;NM_001166266;NM_001166264	Body;Body;Body;Body;Body	1.75E-05	0.02566003
cg02133350	PMS2;PMS2	NM_000535;NR_003085	Body;Body	1.76E-05	0.02566003
cg10857489	SLC12A7	NM_006598	Body	1.77E-05	0.02566003

cg01652215	TPRKB	NM_016058	5'UTR	1.77E-05	0.02566003
cg23475244	NA	NA	NA	1.77E-05	0.02566003
cg13556079	NA	NA	NA	1.78E-05	0.02566003
cg06932451	NA	NA	NA	1.79E-05	0.02566003
cg18246640	NA	NA	NA	1.80E-05	0.02566003
cg03414134	BCL6	NM_001706	5'UTR	1.80E-05	0.02566003
cg02895995	PEX11G	NM_080662	TSS200	1.81E-05	0.02566003
cg20376082	NA	NA	NA	1.81E-05	0.02566003
cg08145495	FUT6;FUT6	NM_001040701;NM_000150	5'UTR;5'UTR	1.82E-05	0.02575763
cg23061412	MIR518A2	NR_030213	Body	1.84E-05	0.02575763
cg00986762	PSMB2;PSMB2	NM_002794;NM_002794	1stExon;5'UTR	1.85E-05	0.02575763
cg15603885	ABRA	NM_139166	1stExon	1.87E-05	0.02575763
cg17169613	RALB	NM_002881	TSS1500	1.87E-05	0.02575763
cg16639311	NA	NA	NA	1.87E-05	0.02575763
cg00464757	NA	NA	NA	1.88E-05	0.02575763
cg23991169	PCGF3	NM_006315	Body	1.88E-05	0.02575763
cg22510662	C5orf33;C5orf33	NM_001085411;NM_153013	Body;Body	1.88E-05	0.02575763
cg17851061	GREB1	NM_014668	Body	1.90E-05	0.02575763
cg11208039	ZDHHC13;ZDHHC13;ZDHHC13;ZDHHC13	NM_001001483;NM_019028;NM_001001483;NM_019028	1stExon;5'UTR;5'UTR;1stExon	1.90E-05	0.02575763
cg19407545	NA	NA	NA	1.90E-05	0.02575763
cg14457132	CAGE1;CAGE1;CAGE1;CAGE1;CAGE1;CAGE1;RIOK1	NM_001170693;NM_001170693;NM_001170692;<br>NM_205864;NM_001170692;NM_205864;NM_031480	1stExon;5'UTR;5'UTR;1stExon;1stExon;5'UTR;<br>TSS200	1.93E-05	0.02590812
cg02668984	PDK2	NM_002611	TSS1500	1.93E-05	0.02590812
cg00090197	TMEM125	NM_144626	Body	1.96E-05	0.02622581
cg23219933	NA	NA	NA	1.97E-05	0.02628317
cg15966151	NR0B2	NM_021969	Body	2.03E-05	0.02689168
cg12652780	PPFIA1;PPFIA1	NM_003626;NM_177423	Body;Body	2.05E-05	0.02689168

cg00402074	GPR116;GPR116	NM_001098518;NM_015234	TSS200;5'UTR	2.06E-05	0.02689168
cg11565042	GPR45	NM_007227	1stExon	2.06E-05	0.02689168
cg06778713	NA	NA	NA	2.06E-05	0.02689168
cg24433586	C17orf56	NM_144679	Body	2.07E-05	0.02689168
cg16925003	PXDN	NM_012293	Body	2.07E-05	0.02689168
cg22312050	NA	NA	NA	2.10E-05	0.02710287
cg20891622	WDR82;MIRLET7G	NM_025222;NR_029660	Body;TSS1500	2.11E-05	0.02714968
cg00638021	COL1A1	NM_000088	Body	2.14E-05	0.0271911
cg11596580	PRMT8;PRMT8	NM_019854;NM_019854	1stExon;5'UTR	2.14E-05	0.0271911
cg17321888	NA	NA	NA	2.14E-05	0.0271911
cg06580551	ZNF662;ZNF662;ZNF662	NM_001134656;NM_207404;NM_207404	TSS200;5'UTR;1stExon	2.15E-05	0.0271911
cg05318427	KCMF1	NM_020122	Body	2.18E-05	0.02752757
cg13701509	NPM1;NPM1;NPM1	NM_002520;NM_199185;NM_001037738	Body;Body;3'UTR	2.19E-05	0.02752757
cg07835232	DENND2A	NM_015689	TSS1500	2.20E-05	0.02752757
cg10505024	KEAP1;KEAP1	NM_203500;NM_012289	Body;Body	2.21E-05	0.02754768
cg19819135	SFRS11	NM_004768	Body	2.26E-05	0.02807133
cg09379601	DNASE2;DNASE2	NM_001375;NM_001375	5'UTR;1stExon	2.27E-05	0.02810405
cg03312366	NA	NA	NA	2.28E-05	0.02819937
cg07031551	ARAP1	NM_001040118	5'UTR	2.32E-05	0.02851273
cg02883247	KIRREL3;KIRREL3	NM_001161707;NM_032531	Body;Body	2.33E-05	0.02857483
cg00882175	NA	NA	NA	2.36E-05	0.02870993
cg12484590	NA	NA	NA	2.36E-05	0.02870993
cg00617292	TNXB	NM_019105	Body	2.37E-05	0.02880934
cg00097968	RPTN	NM_001122965	3'UTR	2.39E-05	0.02886096
cg14464244	MAGI2	NM_012301	TSS200	2.43E-05	0.02920494
cg14840863	COLEC11;COLEC11	NM_024027;NM_199235	Body;Body	2.43E-05	0.02920494

cg16974379	ZNF248	NM_021045	TSS200	2.47E-05	0.02954878
cg13436417	PPAPDC1A	NM_001030059	Body	2.48E-05	0.02957784
cg22627950	TMED4	NM_182547	TSS1500	2.50E-05	0.02962882
cg13129888	EYA3;EYA3	NM_001990;NM_001990	1stExon;5'UTR	2.50E-05	0.02962882
cg03871124	NA	NA	NA	2.51E-05	0.02962882
cg13238876	NA	NA	NA	2.52E-05	0.02965273
cg15985509	ZNF444	NM_018337	Body	2.58E-05	0.03017098
cg24146685	MOGAT3	NM_178176	TSS1500	2.58E-05	0.03017098
cg12438413	RARA;RARA;RARA;RARA	NM_001145301;NM_000964;NM_001024809;NM_001145302	Body;Body;Body;Body	2.61E-05	0.0303575
cg11057205	NA	NA	NA	2.62E-05	0.03038824
cg17460597	NA	NA	NA	2.63E-05	0.03040669
cg03758392	C3orf70	NM_001025266	Body	2.65E-05	0.03043986
cg13265914	REEP4	NM_025232	TSS200	2.65E-05	0.03043986
cg01176329	GABBR1;GABBR1;GABBR1	NM_021904;NM_021903;NM_001470	Body;Body;Body	2.67E-05	0.03049282
cg19240857	LY6G6E;LY6G6E;LY6G6D	NR_003673;NR_024541;NM_021246	TSS1500;TSS1500;TSS200	2.68E-05	0.03049282
cg12739914	NA	NA	NA	2.68E-05	0.03049282
cg11530914	FHOD1;SLC9A5	NM_013241;NM_004594	TSS200;TSS1500	2.71E-05	0.03069211
cg20980271	CLCC1;CLCC1	NM_001048210;NM_015127	TSS1500;TSS1500	2.79E-05	0.03138837
cg02906787	SHC1;SHC1;CKS1B;CKS1B	NM_001130041;NM_003029;NR_024163;NM_001826	TSS1500;TSS1500;Body;Body	2.80E-05	0.03138837
cg13442432	NA	NA	NA	2.80E-05	0.03138837
cg08204369	HN1;HN1;HN1;HN1;HN1;HN1	NM_001002033;NM_001002032;NM_001002032; NM_016185;NM_001002033;NM_016185	5'UTR;5'UTR;1stExon;5'UTR;1stExon;1stExon	2.83E-05	0.03138837
cg02841941	P2RY1	NM_002563	TSS200	2.84E-05	0.03138837
cg24975723	SCARNA9;KIAA1731	NR_002569;NM_033395	TSS1500;Body	2.84E-05	0.03138837
cg21239511	C7orf50;C7orf50;C7orf50	NM_001134395;NM_032350;NM_001134396	Body;Body;Body	2.84E-05	0.03138837
cg24486518	NCAN	NM_004386	Body	2.85E-05	0.03138837
cg22748470	SLC25A2	NM_031947	TSS1500	2.86E-05	0.03138837

cg08438435	APBA2;APBA2	NM_001130414;NM_005503	Body;Body	2.86E-05	0.03138837
cg21939482	C7orf70	NM_001037163	5'UTR	2.88E-05	0.03152276
cg16066544	AOC3	NM_003734	1stExon	2.91E-05	0.03170243
cg17746791	PIM1	NM_002648	TSS200	2.96E-05	0.03187504
cg01332882	ZNFX1	NM_021035	Body	2.96E-05	0.03187504
cg23003225	NA	NA	NA	2.96E-05	0.03187504
cg22249386	MYH13	NM_003802	Body	2.96E-05	0.03187504
cg03705947	GADD45A	NM_001924	Body	3.00E-05	0.03213598
cg09981884	TMEM129;TMEM129	NM_138385;NM_001127266	Body;Body	3.02E-05	0.03224899
cg08274380	GPSM2	NM_013296	TSS200	3.06E-05	0.03252926
cg16276063	SOX2OT	NR_004053	Body	3.06E-05	0.03252926
cg23789977	NA	NA	NA	3.10E-05	0.03269784
cg08370718	DTWD1;DTWD1;DTWD1;DTWD1;C15orf33	NM_020234;NM_001144955;NM_001144955;NM_020234;NM_152647	1stExon;1stExon;5'UTR;5'UTR;TSS200	3.10E-05	0.03269784
cg09878914	BAT1;BAT1	NM_080598;NM_004640	Body;Body	3.12E-05	0.03281818
cg00366594	MIR527	NR_030219	TSS1500	3.15E-05	0.03292777
cg18669135	NA	NA	NA	3.15E-05	0.03292777
cg10211518	POLRMT	NM_005035	TSS200	3.16E-05	0.03292777
cg07612978	C4orf50	NM_207405	TSS1500	3.21E-05	0.03323497
cg15244101	NA	NA	NA	3.21E-05	0.03323497
cg21511036	IRS1	NM_005544	TSS200	3.23E-05	0.03333952
cg02021157	KCNQ1;KCNQ1	NM_000218;NM_181798	Body;Body	3.27E-05	0.03364673
cg01185801	MALAT1	NR_002819	Body	3.28E-05	0.03364673
cg00397871	CCDC129	NM_194300	3'UTR	3.31E-05	0.03392809
cg23593986	MIR499;MYH7B	NR_030223;NM_020884	TSS1500;Body	3.34E-05	0.03398395
cg07127103	MAPK1IP1L	NM_144578	TSS1500	3.36E-05	0.03398395
cg10926047	GFM1	NM_024996	TSS200	3.36E-05	0.03398395

cg20038204	SETBP1	NM_015559	Body	3.39E-05	0.03398395
cg00309965	ECSCR	NM_001077693	TSS200	3.39E-05	0.03398395
cg18934822	SKI	NM_003036	Body	3.40E-05	0.03398395
cg05335473	NA	NA	NA	3.40E-05	0.03398395
cg21687563	TTC28	NM_001145418	Body	3.40E-05	0.03398395
cg22530146	ZNF469	NM_001127464	Body	3.41E-05	0.03398395
cg21864616	NA	NA	NA	3.42E-05	0.03398395
cg17400701	AGAP1;AGAP1	NM_014914;NM_001037131	Body;Body	3.43E-05	0.03398395
cg07312954	DENND4B	NM_014856	Body	3.44E-05	0.03398395
cg20186995	NA	NA	NA	3.46E-05	0.03403432
cg07650554	SEPHS2	NM_012248	TSS200	3.47E-05	0.03403432
cg19881032	SLC16A8	NM_013356	TSS200	3.49E-05	0.03403432
cg12616745	MFSD6	NM_017694	TSS200	3.50E-05	0.03403432
cg11851429	CYB561D1;CYB561D1;CYB561D1;CYB561D1;CYB561D1	NM_001134404;NM_001134402;NM_182580;NM_001134403;NM_001134400	Body;Body;Body;Body;Body	3.50E-05	0.03403432
cg02180424	MDGA2	NM_001113498	Body	3.51E-05	0.03403432
cg07395907	NA	NA	NA	3.52E-05	0.03403432
cg06609415	NA	NA	NA	3.53E-05	0.03403432
cg14771734	ZFYVE16;ZFYVE16	NM_014733;NM_001105251	TSS200;TSS200	3.53E-05	0.03403432
cg02896926	AKNAD1	NM_152763	5'UTR	3.62E-05	0.0345772
cg10901867	NAT8L	NM_178557	Body	3.65E-05	0.0345772
cg16925090	KIAA1377;ANGPTL5	NM_020802;NM_178127	TSS1500;5'UTR	3.66E-05	0.0345772
cg13441112	NA	NA	NA	3.66E-05	0.0345772
cg00160828	NA	NA	NA	3.66E-05	0.0345772
cg19294931	TTBK1	NM_032538	Body	3.66E-05	0.0345772
cg04132851	SSX2IP;SSX2IP;SSX2IP;SSX2IP;SSX2IP	NM_001166294;NM_001166417;NM_001166295;NM_001166293;NM_014021	5'UTR;5'UTR;5'UTR;5'UTR;5'UTR	3.66E-05	0.0345772
cg17422427	RNPEPL1	NM_018226	TSS1500	3.68E-05	0.0345772

cg03133917	RBM33	NM_053043	TSS1500	3.68E-05	0.0345772
cg23784907	PATL1	NM_152716	TSS200	3.71E-05	0.03470613
cg21466107	GLI3	NM_000168	Body	3.72E-05	0.03470613
cg02051981	PAPPA2;PAPPA2	NM_021936;NM_020318	TSS200;TSS200	3.73E-05	0.03470613
cg13551841	EHMT1	NM_024757	Body	3.78E-05	0.03498656
cg20412283	DDAH2	NM_013974	5'UTR	3.80E-05	0.03501512
cg01078248	NDUFA5	NM_005000	Body	3.81E-05	0.03501512
cg17408923	RANBP2	NM_006267	TSS200	3.81E-05	0.03501512
cg06734816	NT5DC1	NM_152729	Body	3.85E-05	0.03510821
cg03811519	NUP214	NM_005085	Body	3.85E-05	0.03510821
cg11752440	DYNC2H1;DYNC2H1	NM_001080463;NM_001377	1stExon;1stExon	3.85E-05	0.03510821
cg19255333	PEMT;PEMT;PEMT	NM_148172;NM_148173;NM_007169	Body;Body;Body	3.88E-05	0.03521699
cg06167524	NA	NA	NA	3.88E-05	0.03521699
cg00598758	TBX20;TBX20	NM_001166220;NM_001077653	Body;Body	3.91E-05	0.03532342
cg22535057	LHFPL4	NM_198560	3'UTR	3.92E-05	0.03532342
cg16466065	LOC388428	NR_027255	Body	3.95E-05	0.03556768
cg06128509	KIF20B	NM_016195	5'UTR	4.01E-05	0.03597064
cg02541778	NA	NA	NA	4.03E-05	0.03597064
cg04324509	PCNP	NM_020357	Body	4.03E-05	0.03597064
cg11181726	NA	NA	NA	4.05E-05	0.03597064
cg10508347	SLC45A1	NM_001080397	1stExon	4.06E-05	0.03597064
cg13948074	C1orf220;C1orf220	NM_207467;NR_033186	TSS1500;TSS1500	4.07E-05	0.03597064
cg12183549	LOC221710;HERV-FRD	NM_001135575;NM_207582	Body;TSS200	4.08E-05	0.03597064
cg13917004	RABAC1	NM_006423	Body	4.08E-05	0.03597064
cg14041194	TRAF2	NM_021138	Body	4.10E-05	0.03606672
cg14099160	SEC63	NM_007214	TSS1500	4.13E-05	0.03620622

cg14028671	NPDC1	NM_015392	Body	4.17E-05	0.0364008
cg11355319	LYSMD3	NM_198273	5'UTR	4.17E-05	0.0364008
cg03584693	RG9MTD1	NM_017819	TSS1500	4.19E-05	0.03642409
cg02194842	NA	NA	NA	4.23E-05	0.03665554
cg01206944	ATP10A	NM_024490	Body	4.29E-05	0.03699582
cg19762797	NA	NA	NA	4.30E-05	0.03699582
cg11024682	SREBF1;SREBF1	NM_004176;NM_001005291	Body;Body	4.30E-05	0.03699582
cg06231385	FOXK1	NM_001037165	Body	4.31E-05	0.03699582
cg04217096	KANK4	NM_181712	TSS1500	4.36E-05	0.03730546
cg03301075	MRGPRE	NM_001039165	TSS1500	4.37E-05	0.03730546
cg00830435	NA	NA	NA	4.44E-05	0.03763528
cg06500161	ABCG1;ABCG1;ABCG1;ABCG1;ABCG1;ABCG1	NM_207628;NM_004915;NM_207174;<br>NM_207627;NM_016818;NM_207629	Body;Body;Body;Body;Body;Body	4.45E-05	0.03763528
cg07036918	SCUBE2;SCUBE2	NM_001170690;NM_020974	TSS200;TSS200	4.46E-05	0.03763528
cg03609216	TRIM15	NM_033229	1stExon	4.46E-05	0.03763528
cg10379653	TBC1D1;PTTG2	NM_015173;NM_006607	Body;1stExon	4.48E-05	0.03763528
cg05104581	ACSF3;ACSF3;ACSF3	NR_023316;NM_001127214;NM_174917	Body;5'UTR;5'UTR	4.49E-05	0.03763528
cg22236894	YTHDF3;YTHDF3	NM_152758;NM_152758	1stExon;5'UTR	4.52E-05	0.03763528
cg21532334	NA	NA	NA	4.52E-05	0.03763528
cg14147105	FAM125A	NM_138401	Body	4.53E-05	0.03763528
cg04266929	NA	NA	NA	4.54E-05	0.03763528
cg09311052	UBE2Q1	NM_017582	TSS1500	4.54E-05	0.03763528
cg15891747	KLHL12;KLHL12	NM_021633;NM_021633	5'UTR;1stExon	4.58E-05	0.03763528
cg12946524	FNDC3B;FNDC3B	NM_022763;NM_001135095	TSS200;TSS1500	4.58E-05	0.03763528
cg10727660	AGAP3	NM_031946	Body	4.59E-05	0.03763528
cg17461336	CYP3A43;CYP3A43;CYP3A43	NM_022820;NM_057096;NM_057095	Body;Body;Body	4.60E-05	0.03763528
cg16163382	NA	NA	NA	4.61E-05	0.03763528

cg02043585	PIGN;PIGN;KIAA1468	NM_176787;NM_012327;NM_020854	5'UTR;5'UTR;TSS1500	4.63E-05	0.03763528
cg11755407	EML3;MTA2	NM_153265;NM_004739	Body;TSS1500	4.63E-05	0.03763528
cg13733010	ZNF716	NM_001159279	3'UTR	4.64E-05	0.03763528
cg17633683	SLC6A18	NM_182632	TSS200	4.66E-05	0.03763528
cg07982899	C4orf22	NM_152770	Body	4.67E-05	0.03763528
cg03045325	MACROD1;FLRT1	NM_014067;NM_013280	Body;TSS200	4.67E-05	0.03763528
cg16814416	TCTEX1D4	NM_001013632	Body	4.68E-05	0.03763528
cg01983759	MED16	NM_005481	3'UTR	4.69E-05	0.03763528
cg07317983	ZBTB20;ZBTB20	NM_015642;NM_001164343	5'UTR;TSS1500	4.69E-05	0.03763528
cg14862791	TTC23L	NM_144725	5'UTR	4.69E-05	0.03763528
cg14842398	KIAA0182	NM_001134473	5'UTR	4.76E-05	0.03774502
cg09173621	ARHGEF7;ARHGEF7;ARHGEF7	NM_001113511;NM_001113512;NM_145735	Body;Body;Body	4.77E-05	0.03774502
cg21790971	FIP1L1;FIP1L1;FIP1L1	NM_030917;NM_001134938;NM_001134937	Body;Body;Body	4.77E-05	0.03774502
cg15026150	ATP8B2	NM_001005855	TSS200	4.78E-05	0.03774502
cg04903691	CXCR5;CXCR5	NM_001716;NM_032966	Body;TSS200	4.79E-05	0.03774502
cg13789711	PHPT1;PHPT1	NM_001135861;NM_014172	TSS200;TSS200	4.79E-05	0.03774502
cg19455335	C8orf58	NM_001013842	Body	4.79E-05	0.03774502
cg08604596	CCDC101	NM_138414	5'UTR	4.79E-05	0.03774502
cg08073153	LEMD2;LEMD2	NM_181336;NM_001143944	Body;TSS200	4.83E-05	0.03797013
cg01334186	PSMB4	NM_002796	Body	4.85E-05	0.03797013
cg20936321	NA	NA	NA	4.87E-05	0.03799396
cg02881186	TRAPPC9;TRAPPC9	NM_001160372;NM_031466	Body;Body	4.87E-05	0.03799396
cg08913530	C10orf129	NM_207321	Body	4.90E-05	0.03810436
cg02716826	SUGT1P1;AQP3	NR_003667;NM_004925	Body;Body	4.94E-05	0.03830621
cg06423822	PTPRN2;PTPRN2;PTPRN2	NM_002847;NM_130842;NM_130843	Body;Body;Body	4.94E-05	0.03830621
cg00522636	DCP2	NM_152624	1stExon	4.97E-05	0.0383309

cg04428700	HOXA13	NM_000522	1stExon	4.98E-05	0.0383309
cg15670865	PLA2G6;PLA2G6	NM_003560;NM_001004426	Body;Body	4.98E-05	0.0383309
cg13397282	DAB2IP;DAB2IP	NM_138709;NM_032552	Body;Body	5.05E-05	0.03872293
cg04226364	MYST2	NM_007067	TSS200	5.05E-05	0.03872293
cg19967421	XCR1;XCR1	NM_005283;NM_001024644	Body;Body	5.07E-05	0.0387637
cg01091261	ADCY9	NM_001116	Body	5.09E-05	0.03877804
cg06199907	MARVELD3	NM_001017967	Body	5.11E-05	0.03877804
cg21104686	NA	NA	NA	5.12E-05	0.03877804
cg02951880	VANGL2	NM_020335	Body	5.12E-05	0.03877804
cg02463166	SPTBN1;SPTBN1	NM_178313;NM_003128	Body;Body	5.15E-05	0.03879009
cg12937438	NA	NA	NA	5.15E-05	0.03879009
cg21099470	ZNF518B	NM_053042	TSS200	5.16E-05	0.03879009
cg09872774	NR2E3;NR2E3	NM_014249;NM_016346	Body;Body	5.17E-05	0.03879009
cg18633432	MSL2	NM_018133	TSS1500	5.18E-05	0.03881808
cg19737664	LRRC56	NM_198075	TSS200	5.22E-05	0.03900233
cg00160522	FGFRL1;FGFRL1;FGFRL1	NM_001004358;NM_001004356;NM_021923	Body;Body;Body	5.24E-05	0.039058
cg14650228	ZC3H3	NM_015117	Body	5.29E-05	0.03933022
cg05567435	METTL7B	NM_152637	TSS1500	5.30E-05	0.03933022
cg24059022	SLC12A7	NM_006598	Body	5.33E-05	0.03952712
cg01243823	NOD2	NM_022162	Body	5.36E-05	0.03960058
cg00310201	ZAP70;ZAP70	NM_001079;NM_207519	Body;Body	5.38E-05	0.03960058
cg14054990	KRTAP19-5	NM_181611	TSS200	5.38E-05	0.03960058
cg19853194	VAPA;VAPA	NM_194434;NM_003574	TSS1500;TSS1500	5.39E-05	0.03960058
cg20566449	RASA3	NM_007368	Body	5.40E-05	0.03960058
cg24044651	C1orf128	NM_020362	Body	5.42E-05	0.0396528
cg19385725	NPC1	NM_000271	Body	5.43E-05	0.03966414

cg04362790	NA	NA	NA	5.45E-05	0.03972649
cg15044954	C7orf63;C7orf63	NM_001039706;NM_001160138	TSS200;TSS200	5.49E-05	0.03992478
cg05978306	MYO1C;MYO1C;MYO1C	NM_001080779;NM_033375;NM_001080950	Body;Body;Body	5.51E-05	0.03995238
cg23402821	NA	NA	NA	5.54E-05	0.03995238
cg16610810	ITGA9	NM_002207	Body	5.55E-05	0.03995238
cg16522993	NA	NA	NA	5.56E-05	0.03995238
cg20136959	CDADC1	NM_030911	TSS200	5.57E-05	0.03995238
cg18906002	KIAA1688	NM_025251	Body	5.58E-05	0.03995238
cg04661591	NA	NA	NA	5.58E-05	0.03995238
cg09299307	CCNH	NM_001239	TSS200	5.59E-05	0.03995238
cg09350880	DPF3	NM_012074	3'UTR	5.61E-05	0.03997204
cg05207943	NA	NA	NA	5.62E-05	0.03997204
cg07936541	ANKRD36B	NM_025190	1stExon	5.63E-05	0.03997204
cg09650907	FAM104A;FAM104A	NM_032837;NM_001098832	Body;Body	5.65E-05	0.03997204
cg11973777	NA	NA	NA	5.66E-05	0.03997204
cg21211680	NA	NA	NA	5.67E-05	0.03997204
cg19500057	STRN3;STRN3;MIR624	NM_001083893;NM_014574;NR_030354	Body;Body;TSS1500	5.68E-05	0.03997204
cg15761814	CCDC138	NM_144978	Body	5.69E-05	0.03997204
cg20418501	NA	NA	NA	5.74E-05	0.04010174
cg06733311	PHF1;PHF1;PHF1	NR_027692;NM_002636;NM_024165	Body;Body;Body	5.74E-05	0.04010174
cg08862564	CREB1;CREB1	NM_134442;NM_004379	5'UTR;5'UTR	5.76E-05	0.04010174
cg03694795	SC65	NM_006455	Body	5.77E-05	0.04010174
cg11235741	MEX3B	NM_032246	Body	5.81E-05	0.04010174
cg18100830	NA	NA	NA	5.85E-05	0.04010174
cg12243375	GUCY1A2	NM_000855	TSS200	5.85E-05	0.04010174
cg07904028	PPP2R2C;PPP2R2C	NM_181876;NM_020416	Body;Body	5.86E-05	0.04010174

cg19225280	TAF11	NM_005643	3'UTR	5.86E-05	0.04010174
cg20032723	DCPS	NM_014026	1stExon	5.86E-05	0.04010174
cg02263813	MT1A;MT1A	NM_005946;NM_005946	1stExon;5'UTR	5.88E-05	0.04010174
cg08251160	MGC72080	NR_002822	Body	5.88E-05	0.04010174
cg22346032	NA	NA	NA	5.91E-05	0.04010174
cg03678860	TBCD	NM_005993	Body	5.91E-05	0.04010174
cg24624173	SLC22A11	NM_018484	Body	5.92E-05	0.04010174
cg12653788	NA	NA	NA	5.92E-05	0.04010174
cg09869950	FGGY;FGGY	NM_018291;NM_001113411	5'UTR;5'UTR	5.93E-05	0.04010174
cg04767756	EMP2	NM_001424	5'UTR	5.94E-05	0.04010174
cg05639679	KIAA1522	NM_020888	Body	5.94E-05	0.04010174
cg18420708	EPHX3;EPHX3	NM_024794;NM_001142886	Body;Body	5.95E-05	0.04010174
cg18350497	C11orf54	NM_014039	5'UTR	5.97E-05	0.04010174
cg03708092	NA	NA	NA	5.98E-05	0.04010174
cg11342452	NKX6-2	NM_177400	TSS1500	5.99E-05	0.04010174
cg23095383	PTPRC;PTPRC;PTPRC;PTPRC	NM_080922;NM_002838;NM_080923;NM_080921	Body;Body;Body;Body	5.99E-05	0.04010174
cg21774377	UHRF1BP1	NM_017754	1stExon	6.00E-05	0.04010174
cg20011604	TUBA3C	NM_006001	Body	6.03E-05	0.04017582
cg18365406	LPPR2;LPPR2	NM_022737;NM_001170635	Body;Body	6.04E-05	0.04017582
cg17204834	ZNF589	NM_016089	TSS200	6.05E-05	0.04017582
cg06963308	NA	NA	NA	6.08E-05	0.04024596
cg14420670	NA	NA	NA	6.09E-05	0.04024596
cg03157738	KANK2	NM_001136191	5'UTR	6.09E-05	0.04024596
cg07823492	HOXB1	NM_002144	1stExon	6.10E-05	0.04024596
cg16890600	NA	NA	NA	6.18E-05	0.04061619
cg07758528	PARP8	NM_024615	Body	6.18E-05	0.04061619

cg14138312	KDM4C;KDM4C;KDM4C;KDM4C	NM_015061;NM_001146694;NM_001146695;NM_001146696	Body;Body;Body;Body	6.27E-05	0.0410154
cg06873218	PDGFB;PDGFB	NM_002608;NM_033016	Body;TSS1500	6.28E-05	0.0410154
cg24536827	NA	NA	NA	6.28E-05	0.0410154
cg18391611	SBK2	NM_001101401	Body	6.30E-05	0.04107536
cg01973725	RNU5E;RNU5D;ZCCHC9;ZCCHC9;ZCCHC9	NR_002754;NR_002755;NM_032280;NM_001131036;NM_001131035	Body;Body;3'UTR;3'UTR;3'UTR	6.32E-05	0.04107536
cg24250234	NA	NA	NA	6.33E-05	0.04107536
cg12593303	SPTBN1;SPTBN1	NM_178313;NM_003128	TSS200;Body	6.38E-05	0.04137592
cg12738349	MIF	NM_002415	TSS1500	6.42E-05	0.04151142
cg10790887	INPP5A	NM_005539	Body	6.43E-05	0.04152049
cg04661747	NA	NA	NA	6.47E-05	0.04157528
cg20248822	C1orf151	NM_001032363	TSS1500	6.48E-05	0.04157528
cg18511558	ARHGEF10	NM_014629	Body	6.49E-05	0.04157528
cg01093065	PLEKHA4;PLEKHA4	NM_001161354;NM_020904	TSS1500;TSS1500	6.53E-05	0.04157528
cg12340219	GPR123	NM_001083909	Body	6.54E-05	0.04157528
cg03195665	FPR2;FPR2	NM_001005738;NM_001462	5'UTR;TSS1500	6.55E-05	0.04157528
cg24552424	SLCO5A1;SLCO5A1;SLCO5A1	NM_001146008;NM_030958;NM_001146009	3'UTR;Body;Body	6.55E-05	0.04157528
cg23319790	MIR519D;MIR517A	NR_030202;NR_030201	TSS1500;TSS200	6.57E-05	0.04157528
cg06954660	HLA-DOA	NM_002119	Body	6.59E-05	0.04157528
cg20451986	NA	NA	NA	6.59E-05	0.04157528
cg08964024	NA	NA	NA	6.59E-05	0.04157528
cg09564133	MXD3;MXD3;MXD3;MXD3	NM_031300;NM_001142935;NM_031300;NM_001142935	1stExon;1stExon;5'UTR;5'UTR	6.61E-05	0.04157528
cg17362483	NA	NA	NA	6.62E-05	0.04157528
cg13944397	NA	NA	NA	6.63E-05	0.04157528
cg14750501	CCNE1;CCNE1;CCNE1	NM_057182;NM_001238;NM_001238	TSS1500;5'UTR;1stExon	6.64E-05	0.04157528
cg18911817	NKD2	NM_033120	Body	6.65E-05	0.04157528
cg16300033	ANKRD30A	NM_052997	TSS1500	6.69E-05	0.04157528

cg17588812	KIF1A	NM_004321	Body	6.69E-05	0.04157528
cg22628240	NA	NA	NA	6.70E-05	0.04157528
cg01721822	TMEM88B	NM_001146685	TSS1500	6.73E-05	0.04157528
cg14964327	NA	NA	NA	6.73E-05	0.04157528
cg11791144	NRG1	NM_013962	Body	6.74E-05	0.04157528
cg19701416	NA	NA	NA	6.74E-05	0.04157528
cg05397362	NA	NA	NA	6.74E-05	0.04157528
cg21151017	NA	NA	NA	6.74E-05	0.04157528
cg23413289	FAT1	NM_005245	Body	6.77E-05	0.04159311
cg05233324	NA	NA	NA	6.77E-05	0.04159311
cg21367769	SCGN	NM_006998	TSS1500	6.78E-05	0.04159311
cg00008819	C4orf44;C4orf44	NM_001042690;NM_001012982	3'UTR;Body	6.80E-05	0.04161751
cg14476101	PHGDH	NM_006623	Body	6.81E-05	0.04161751
cg04948956	NA	NA	NA	6.88E-05	0.04198003
cg23226387	TACO1	NM_016360	TSS1500	6.90E-05	0.04200428
cg01374398	SCAP	NM_012235	5'UTR	6.93E-05	0.04211344
cg16956456	C5orf41;C5orf41;C5orf41;C5orf41;C5orf41;C5orf41	NM_001168394;NM_153607;NM_001168394;NM_001168393;NM_001168393;NM_153607	1stExon;1stExon;5'UTR;1stExon;5'UTR;5'UTR	6.95E-05	0.04218603
cg21120176	LOC285780	NR_026970	Body	6.99E-05	0.04231198
cg08695707	C1orf53	NM_001024594	1stExon	7.00E-05	0.04231198
cg02078896	MACROD2;MACROD2	NM_080676;NM_001033087	Body;TSS1500	7.05E-05	0.04253703
cg15537788	SIPA1L3	NM_015073	3'UTR	7.06E-05	0.04254987
cg22957121	MGAT4C	NM_013244	5'UTR	7.12E-05	0.04283115
cg19116956	NA	NA	NA	7.14E-05	0.04284437
cg04423542	TRIM39;HCG18;HCG18;TRIM39	NM_021253;NR_024052;NR_024053;NM_172016	TSS1500;Body;Body;TSS1500	7.17E-05	0.04298482
cg15965155	NA	NA	NA	7.21E-05	0.04310131
cg13088902	NA	NA	NA	7.24E-05	0.04320446

cg02765496	FAM122A;PIP5K1B	NM_138333;NM_003558	TSS1500;5'UTR	7.30E-05	0.04337925
cg05947499	TPCN1;TPCN1	NM_001143819;NM_017901	Body;Body	7.30E-05	0.04337925
cg24420395	DUSP4;DUSP4	NM_001394;NM_057158	Body;TSS1500	7.31E-05	0.04337925
cg14007221	FRMPD1	NM_014907	5'UTR	7.32E-05	0.04337925
cg25233569	RHOBTB1;RHOBTB1;RHOBTB1;RHOBTB1;RHOBTB1	NR_024555;NM_014836;NM_014836;NR_024554;NR_024556	Body;1stExon;5'UTR;Body;Body	7.38E-05	0.04363211
cg06677352	PMS2L2;STAG3L3;STAG3L3	NR_003614;NM_001013739;NM_001013739	TSS1500;5'UTR;1stExon	7.40E-05	0.04363211
cg09391949	JPH3	NM_020655	3'UTR	7.44E-05	0.04367326
cg13554030	LSM2	NM_021177	Body	7.46E-05	0.04367326
cg17780691	MYH3	NM_002470	Body	7.46E-05	0.04367326
cg09198463	ATL2;ATL2;ATL2	NM_022374;NM_001135673;NR_024191	Body;Body;Body	7.47E-05	0.04367326
cg21583412	NUFIP2	NM_020772	TSS1500	7.49E-05	0.04367326
cg01489057	DMBT1;DMBT1;DMBT1	NM_007329;NM_004406;NM_017579	Body;Body;Body	7.50E-05	0.04367326
cg24162465	NA	NA	NA	7.51E-05	0.04367326
cg11333007	TMOD3;TMOD3	NM_014547;NM_014547	5'UTR;1stExon	7.51E-05	0.04367326
cg08814206	PPT2;PPT2	NM_005155;NM_138717	Body;Body	7.53E-05	0.04368054
cg12884982	JPH1	NM_020647	Body	7.53E-05	0.04368054
cg06307212	EGFL8	NM_030652	Body	7.55E-05	0.04370427
cg00894870	MRTO4;KIAA0090	NM_016183;NM_015047	Body;TSS1500	7.58E-05	0.04377161
cg21654058	CCDC137	NM_199287	3'UTR	7.61E-05	0.04377253
cg05864433	AMZ1	NM_133463	5'UTR	7.61E-05	0.04377253
cg21273856	NCOA7;NCOA7;NCOA7;NCOA7	NM_181782;NM_001122842;NM_181782;NM_001122842	5'UTR;5'UTR;1stExon;1stExon	7.61E-05	0.04377253
cg13896328	PTPRU;PTPRU;PTPRU	NM_005704;NM_133178;NM_133177	3'UTR;3'UTR;3'UTR	7.65E-05	0.04383002
cg10187692	VPS13B;VPS13B	NM_017890;NM_152564	Body;Body	7.66E-05	0.04383002
cg08788291	RECQL5;LOC643008	NM_004259;NM_001162995	Body;TSS1500	7.66E-05	0.04383002
cg20303815	NA	NA	NA	7.70E-05	0.04396392
cg02866076	NA	NA	NA	7.73E-05	0.04399808

cg03900195	CHAF1B	NM_005441	5'UTR	7.74E-05	0.04399808
cg14253896	NA	NA	NA	7.75E-05	0.04399808
cg25244200	NA	NA	NA	7.76E-05	0.04399808
cg23978242	DIP2C	NM_014974	Body	7.78E-05	0.04401179
cg13135459	LOC387646	NR_003525	TSS200	7.79E-05	0.04401179
cg00588689	NA	NA	NA	7.80E-05	0.04401179
cg17767933	NA	NA	NA	7.82E-05	0.04401179
cg02393907	ACTR3C	NM_001164458	5'UTR	7.82E-05	0.04401179
cg08722720	AVEN;CHRM5	NM_020371;NM_012125	TSS1500;5'UTR	7.85E-05	0.04403307
cg07016356	CXCR1	NM_000634	TSS1500	7.86E-05	0.04403307
cg16053745	C3orf43	NM_001077657	Body	7.87E-05	0.04403307
cg13462275	TCP10	NM_004610	TSS1500	7.88E-05	0.04403307
cg09920427	MORG1;MORG1;MAN2B1;MORG1	NM_001099737;NM_001099737;NM_000528;NR_029375	1stExon;5'UTR;TSS200;Body	7.91E-05	0.04403307
cg02956660	HSPG2	NM_005529	Body	7.93E-05	0.04403307
cg16209873	OR52W1	NM_001005178	TSS1500	7.93E-05	0.04403307
cg08015507	NCKIPSD;NCKIPSD	NM_016453;NM_184231	Body;Body	7.93E-05	0.04403307
cg00007800	NA	NA	NA	7.94E-05	0.04403307
cg25214900	SYT1;SYT1;SYT1	NM_005639;NM_001135805;NM_001135806	Body;Body;Body	7.99E-05	0.04422224
cg05063097	ZNF423	NM_015069	TSS1500	8.01E-05	0.04422224
cg18181703	SOCS3	NM_003955	Body	8.02E-05	0.04422224
cg06293170	NA	NA	NA	8.04E-05	0.04424017
cg02891801	SCNN1A;SCNN1A;SCNN1A	NM_001159575;NM_001038;NM_001159576	Body;Body;Body	8.05E-05	0.04424017
cg17126142	NA	NA	NA	8.06E-05	0.04424017
cg02581828	NA	NA	NA	8.08E-05	0.04424017
cg08577693	NUTF2	NM_005796	Body	8.08E-05	0.04424017
cg13659329	NA	NA	NA	8.10E-05	0.04427962

cg18219432	PTPRN2;PTPRN2;PTPRN2	NM_002847;NM_130842;NM_130843	Body;Body;Body	8.16E-05	0.04443359
cg09174741	THSD1;THSD1	NM_199263;NM_018676	TSS1500;TSS1500	8.17E-05	0.04443359
cg02412123	SIGLEC9;SIGLEC9	NM_014441;NM_014441	5'UTR;1stExon	8.17E-05	0.04443359
cg04678715	FANK1	NM_145235	Body	8.19E-05	0.04443359
cg02894226	ITPA;ITPA	NM_181493;NM_033453	Body;Body	8.23E-05	0.04461203
cg20168964	TMEM132D	NM_133448	Body	8.25E-05	0.04464195
cg13875763	NA	NA	NA	8.29E-05	0.04479279
cg09764443	POU2F3	NM_014352	TSS1500	8.35E-05	0.04499695
cg07022307	ATP1A4;ATP1A4	NM_001001734;NM_144699	TSS1500;Body	8.36E-05	0.04499695
cg06788172	DMXL2	NM_015263	TSS200	8.39E-05	0.04508467
cg18181431	NA	NA	NA	8.40E-05	0.04508467
cg21457856	NA	NA	NA	8.42E-05	0.04514076
cg05086567	MPDU1;MPDU1	NM_004870;NR_024603	TSS1500;TSS1500	8.45E-05	0.04515042
cg11668638	PPP2R5D;PPP2R5D;PPP2R5D;PPP2R5D;PPP2R5D;PPP2R5D	NM_180977;NM_180976;NM_180976;NM_006245;NM_006245;NM_180977	5'UTR;5'UTR;1stExon;5'UTR;1stExon;1stExon	8.45E-05	0.04515042
cg06330593	KRT34	NM_021013	Body	8.49E-05	0.04530231
cg13426340	NA	NA	NA	8.51E-05	0.04532096
cg09864156	SUPV3L1	NM_003171	TSS1500	8.53E-05	0.04533359
cg05761882	CAPN2;CAPN2	NM_001748;NM_001146068	Body;Body	8.54E-05	0.04533359
cg06115835	SH3RF3	NM_001099289	Body	8.58E-05	0.04549325
cg05063087	C10orf75	NR_026762	TSS200	8.59E-05	0.04549325
cg17951368	NUCB2	NM_005013	TSS200	8.63E-05	0.04561776
cg03640568	DDX17;DDX17	NM_006386;NM_001098504	TSS1500;TSS1500	8.65E-05	0.04561776
cg02021544	MYLK3	NM_182493	TSS1500	8.67E-05	0.04561776
cg05626927	NA	NA	NA	8.70E-05	0.04561776
cg04993564	NSMAF;NSMAF	NM_003580;NM_001144772	Body;TSS200	8.70E-05	0.04561776
cg04026699	NA	NA	NA	8.71E-05	0.04561776

cg11869499	POLG;POLG	NM_001126131;NM_002693	Body;Body	8.73E-05	0.04561776
cg07189582	SEC24B;SEC24B	NM_006323;NM_001042734	Body;Body	8.74E-05	0.04561776
cg05353415	GLI3	NM_000168	5'UTR	8.74E-05	0.04561776
cg21546837	NA	NA	NA	8.76E-05	0.04561776
cg19504496	RBM42	NM_024321	TSS200	8.76E-05	0.04561776
cg06273763	MYO1E;MYO1E	NM_004998;NM_004998	5'UTR;1stExon	8.83E-05	0.04588118
cg19418922	EXT2;EXT2	NM_000401;NM_207122	3'UTR;3'UTR	8.85E-05	0.04588118
cg02655824	RPS6KA2	NM_001006932	Body	8.87E-05	0.04588118
cg06134974	CSRNP2	NM_030809	TSS1500	8.87E-05	0.04588118
cg23624929	NA	NA	NA	8.91E-05	0.04600861
cg14249922	GADD45G	NM_006705	3'UTR	8.96E-05	0.04609631
cg16815882	KIAA0319L	NM_024874	Body	8.97E-05	0.04609631
cg08846459	NA	NA	NA	8.98E-05	0.04609631
cg12853981	TOMM20L	NM_207377	1stExon	8.98E-05	0.04609631
cg13086025	KIAA1967;KIAA1967	NM_021174;NM_199205	Body;Body	9.01E-05	0.0461738
cg24677472	NA	NA	NA	9.02E-05	0.0461738
cg00658449	BEYLA;BEYLA	NR_027012;NR_027013	TSS1500;TSS1500	9.07E-05	0.04630641
cg20111269	BTBD11	NM_001018072	Body	9.08E-05	0.04630641
cg06548214	NPEPL1	NM_024663	Body	9.09E-05	0.04630641
cg03890538	RPTOR;RPTOR	NM_020761;NM_001163034	3'UTR;3'UTR	9.13E-05	0.04640457
cg19526076	NA	NA	NA	9.13E-05	0.04640457
cg05072951	CAPN1;CAPN1	NM_005186;NM_005186	1stExon;5'UTR	9.17E-05	0.04646561
cg06611444	NA	NA	NA	9.17E-05	0.04646561
cg10981770	C1orf122;YRDC;C1orf122	NM_198446;NM_024640;NM_001142726	TSS1500;Body;TSS1500	9.20E-05	0.04652489
cg10996143	FKBP1A;FKBP1A	NM_000801;NM_054014	TSS200;TSS200	9.25E-05	0.04669819
cg03648631	UBE2D3;UBE2D3;UBE2D3;UBE2D3; UBE2D3;UBE2D3;UBE2D3;UBE2D3;UBE2D3	NM_181887;NM_181891;NM_181889;NM_181888; NM_181892;NM_181893;NM_003340;NM_181890;NM_181886	TSS1500;TSS1500;TSS200;TSS1500;TSS1500;B ody;TSS1500;5'UTR;TSS1500	9.27E-05	0.04669819

cg14542646	NA	NA	NA	9.30E-05	0.04669819
cg07766948	CUEDC2	NM_024040	5'UTR	9.32E-05	0.04669819
cg01258793	MAD1L1;MAD1L1;MAD1L1	NM_003550;NM_001013837;NM_001013836	Body;Body;Body	9.32E-05	0.04669819
cg17562763	NUDT8	NM_181843	TSS1500	9.33E-05	0.04669819
cg06332304	CTBP2;CTBP2	NM_001083914;NM_001329	5'UTR;5'UTR	9.33E-05	0.04669819
cg08540945	NA	NA	NA	9.35E-05	0.04669819
cg05304037	PRR12;PRRG2	NM_020719;NM_000951	TSS1500;3'UTR	9.36E-05	0.04669819
cg10089324	EPS15	NM_001981	Body	9.47E-05	0.04721343
cg13695383	AGFG2	NM_006076	Body	9.50E-05	0.04727339
cg19775406	MIS12;MIS12;DERL2	NM_024039;NM_024039;NM_016041	5'UTR;1stExon;TSS1500	9.55E-05	0.04743654
cg25188165	NA	NA	NA	9.57E-05	0.04750452
cg21950037	NA	NA	NA	9.62E-05	0.04763259
cg03443922	CBLL1;CBLL1	NM_024814;NR_024199	Body;Body	9.65E-05	0.04763259
cg10577346	RPL27A;RPL27A	NM_000990;NM_000990	5'UTR;1stExon	9.66E-05	0.04763259
cg05608508	NTAN1	NM_173474	Body	9.68E-05	0.04763259
cg03797768	NA	NA	NA	9.70E-05	0.04763259
cg01789926	C16orf91	NM_001010878	Body	9.72E-05	0.04763259
cg21515243	RIOK3;RIOK3	NM_003831;NM_003831	1stExon;5'UTR	9.72E-05	0.04763259
cg05571558	ATP2B2;MIR885;ATP2B2	NM_001683;NR_030614;NM_001001331	Body;TSS200;Body	9.72E-05	0.04763259
cg15128334	TMEM41A	NM_080652	Body	9.74E-05	0.04763259
cg16098340	MIR548C;RASSF3	NR_030347;NM_178169	TSS1500;Body	9.74E-05	0.04763259
cg18342900	C16orf87	NM_001001436	1stExon	9.75E-05	0.04763259
cg16692534	FOXK2	NM_004514	Body	9.77E-05	0.04763326
cg12456777	SNORD42B;RPL23A	NR_000013;NM_000984	TSS1500;1stExon	9.78E-05	0.04763326
cg09468777	PDE6B;PDE6B;PDE6B	NM_000283;NM_001145291;NM_001145292	Body;Body;Body	9.82E-05	0.04769851
cg18762795	NA	NA	NA	9.86E-05	0.04769851

cg13057368	SSB;SSB	NM_003142;NM_003142	5'UTR;1stExon	9.86E-05	0.04769851
cg07433154	NA	NA	NA	9.87E-05	0.04769851
cg07980405	ZBTB12	NM_181842	TSS1500	9.89E-05	0.04769851
cg12610079	C1orf110	NM_178550	TSS1500	9.94E-05	0.04769851
cg07960624	SAMD12	NM_001101676	3'UTR	9.94E-05	0.04769851
cg24969222	NA	NA	NA	9.94E-05	0.04769851
cg04317962	NA	NA	NA	9.94E-05	0.04769851
cg00474854	GNAL;GNAL;GNAL	NM_001142339;NM_182978;NM_002071	Body;Body;Body	9.97E-05	0.04769851
cg09234616	WT1;WT1;WT1;WT1	NM_024425;NM_024426;NM_000378;NM_024424	Body;Body;Body;Body	9.97E-05	0.04769851
cg24263283	MYO1E	NM_004998	Body	9.98E-05	0.04769851
cg11332951	NA	NA	NA	9.98E-05	0.04769851
cg14563732	TSNARE1	NM_145003	5'UTR	9.99E-05	0.04769851
cg11547486	NET1	NM_001047160	TSS200	0.00010039	0.04786349
cg01335566	CHD5	NM_015557	Body	0.00010064	0.04791824
cg15358743	SEC11C	NM_033280	TSS200	0.00010088	0.04796471
cg17586419	TOP1P2;PIWIL3	NR_001283;NM_001008496	TSS1500;5'UTR	0.00010118	0.04798878
cg02194046	NA	NA	NA	0.00010135	0.04798878
cg20217938	NA	NA	NA	0.00010164	0.04798878
cg22730398	NA	NA	NA	0.00010175	0.04798878
cg10082357	ATP6V1F	NM_004231	3'UTR	0.00010202	0.04798878
cg15305352	NA	NA	NA	0.00010212	0.04798878
cg10126022	NA	NA	NA	0.00010254	0.04798878
cg23330006	NUAK1	NM_014840	Body	0.00010282	0.04798878
cg17740822	DUSP6;DUSP6	NM_001946;NM_022652	Body;Body	0.00010282	0.04798878
cg01264963	OR10G4	NM_001004462	TSS1500	0.00010284	0.04798878
cg01261044	SRP54;SRP54	NM_003136;NM_001146282	TSS200;TSS200	0.0001029	0.04798878

cg18651192	NA	NA	NA	0.00010316	0.04798878
cg17367356	MAPK8IP1	NM_005456	TSS200	0.00010318	0.04798878
cg06900676	KIAA1841;KIAA1841	NM_001129993;NM_032506	5'UTR;TSS200	0.00010353	0.04798878
cg09887867	NA	NA	NA	0.0001037	0.04798878
cg06883956	MEGF6;MIR551A	NM_001409;NR_030277	Body;Body	0.00010379	0.04798878
cg11875044	NA	NA	NA	0.00010384	0.04798878
cg00143700	RCBTB2	NM_001268	5'UTR	0.00010394	0.04798878
cg12096988	ZNF264	NM_003417	TSS200	0.00010416	0.04798878
cg15929201	RNASEN;C5orf22;RNASEN	NM_013235;NM_018356;NM_001100412	5'UTR;TSS1500;5'UTR	0.00010426	0.04798878
cg08571347	NA	NA	NA	0.00010427	0.04798878
cg15312298	FAM84B	NM_174911	TSS1500	0.00010434	0.04798878
cg12639422	UGP2;UGP2;UGP2	NM_006759;NM_006759;NM_001001521	1stExon;5'UTR;5'UTR	0.00010439	0.04798878
cg11615347	ENPP7	NM_178543	Body	0.00010445	0.04798878
cg01903185	FGFR3;FGFR3;FGFR3	NM_001163213;NM_000142;NM_022965	Body;Body;Body	0.00010475	0.04798878
cg03820205	VDAC2	NM_003375	TSS200	0.00010506	0.04798878
cg11690896	BIRC2	NM_001166	TSS200	0.00010517	0.04798878
cg16542646	NA	NA	NA	0.00010532	0.04798878
cg12455797	TCERG1L	NM_174937	Body	0.00010536	0.04798878
cg13463033	SEC16A	NM_014866	Body	0.00010544	0.04798878
cg01764559	NA	NA	NA	0.00010586	0.04810262
cg22129122	NA	NA	NA	0.00010597	0.04810262
cg19741408	ZNF326;LOC492303;ZNF326	NM_182975;NR_002830;NM_182976	TSS1500;Body;TSS1500	0.00010613	0.04811256
cg05053688	S1PR1	NM_001400	5'UTR	0.00010635	0.04814618
cg20149840	HDAC4	NM_006037	Body	0.00010677	0.04827524
cg15888741	RPP38;RPP38;RPP38;C10orf111;<br>RPP38;C10orf111;RPP38;RPP38	NM_006414;NM_001097590;NM_001097590;<br>NM_153244;NM_183005;NM_153244;NM_183005;NM_006414	1stExon;5'UTR;1stExon;1stExon;5'UTR;5'UTR;<br>1stExon;5'UTR	0.00010699	0.04830948
cg17500329	TRAPPC9;TRAPPC9	NM_001160372;NM_031466	Body;Body	0.00010735	0.04831215

cg04446992	NA	NA	NA	0.0001074	0.04831215
cg09755102	S100PBP;YARS;YARS;S100PBP	NM_022753;NM_003680;NM_003680;NM_001017406	5'UTR;1stExon;5'UTR;5'UTR	0.00010769	0.04831215
cg04608330	NELL2;NELL2;NELL2;NELL2;NELL2	NM_001145110;NM_001145107;NM_006159; NM_001145108;NM_001145109	Body;Body;Body;Body;1stExon	0.00010788	0.04831215
cg23767977	KRT71	NM_033448	TSS1500	0.0001079	0.04831215
cg08039585	C1orf159	NM_017891	Body	0.00010818	0.04831215
cg23661013	HNF1A	NM_000545	TSS1500	0.00010828	0.04831215
cg01965508	PIGR	NM_002644	TSS1500	0.00010836	0.04831215
cg04887124	NA	NA	NA	0.00010839	0.04831215
cg06451949	NA	NA	NA	0.00010841	0.04831215
cg16020249	AHDC1	NM_001029882	TSS1500	0.00010865	0.04835462
cg12593793	NA	NA	NA	0.00010903	0.04845816
cg04138756	SPRR3;SPRR3	NM_001097589;NM_005416	TSS1500;TSS1500	0.00010952	0.048616
cg02553489	SQLE;SQLE	NM_003129;NM_003129	1stExon;5'UTR	0.00010985	0.04869943
cg00334821	LIMK1	NM_002314	Body	0.00011024	0.04878772
cg07723459	NA	NA	NA	0.00011053	0.04878772
cg03209809	CHCHD6	NM_032343	TSS1500	0.0001106	0.04878772
cg09596336	ZEB2;ZEB2;ZEB2	NR_033258;NM_014795;NM_001171653	Body;Body;Body	0.00011063	0.04878772
cg19484141	SMUG1	NM_014311	Body	0.00011107	0.04892208
cg11911399	SLC25A24;SLC25A24	NM_013386;NM_013386	1stExon;5'UTR	0.00011135	0.04898
cg12664633	ZBTB22;ZBTB22;ZBTB22	NM_001145338;NM_005453;NM_001145338	5'UTR;TSS200;1stExon	0.00011171	0.04907764
cg18160291	HHAT;HHAT;HHAT;HHAT;HHAT;HHAT	NM_001170580;NM_001170564;NM_001170587; NM_001170588;NM_001122834;NM_018194	TSS1500;5'UTR;TSS200;TSS200;5'UTR;TSS200	0.00011197	0.04912855
cg25020232	NA	NA	NA	0.00011221	0.04913789
cg04621069	ECE1;ECE1;ECE1;ECE1	NM_001113347;NM_001397;NM_001113348;NM_001113349	3'UTR;3'UTR;3'UTR;3'UTR	0.00011228	0.04913789
cg24968629	CELSR1	NM_014246	Body	0.00011262	0.04922124
cg16476048	C10orf122	NM_001128202	TSS1500	0.00011364	0.04948847
cg08266945	C2orf77;C2orf77;PHOSPHO2;KLHL23	NM_001085447;NM_001085447;NM_001008489;NM_144711	5'UTR;1stExon;TSS200;TSS200	0.00011386	0.04948847

cg20655342	NA	NA	NA	0.00011393	0.04948847
cg22716292	NA	NA	NA	0.00011411	0.04948847
cg09896298	DUPD1	NM_001003892	TSS200	0.00011431	0.04948847
cg23976179	NA	NA	NA	0.00011442	0.04948847
cg24288751	NA	NA	NA	0.00011445	0.04948847
cg13749939	SLC25A22	NM_024698	TSS1500	0.0001145	0.04948847
cg13611636	ZMAT4;ZMAT4	NM_001135731;NM_024645	5'UTR;5'UTR	0.00011454	0.04948847
cg19203575	ZNF323;ZKSCAN3;ZNF323	NM_001135215;NM_024493;NM_145909	5'UTR;5'UTR;TSS200	0.00011484	0.04955538
cg09198085	AK7	NM_152327	Body	0.00011531	0.04966023
cg18006626	NA	NA	NA	0.00011551	0.04966023
cg04786791	RRP15	NM_016052	TSS200	0.00011566	0.04966023
cg22583147	C3orf77	NM_001145030	Body	0.00011587	0.04966023
cg08104402	IFT140;CRAMP1L	NM_014714;NM_020825	TSS1500;TSS1500	0.00011624	0.04966023
cg01141459	HORMAD2;HORMAD2	NM_152510;NM_152510	1stExon;5'UTR	0.00011626	0.04966023
cg21766592	SLC1A5;SLC1A5;SLC1A5;SLC1A5	NM_001145145;NM_001145145;NM_005628;NM_001145144	1stExon;5'UTR;Body;5'UTR	0.00011628	0.04966023
cg23592392	NA	NA	NA	0.00011638	0.04966023
cg16705446	APBA3	NM_004886	Body	0.00011639	0.04966023
cg01372058	SF3B2	NM_006842	TSS1500	0.00011657	0.04966816
cg04358264	CYP3A4	NM_017460	3'UTR	0.0001167	0.04966816
cg07797833	NA	NA	NA	0.00011753	0.04995858

Table S2: Selection of 478 CpGs after comparing individuals with HOMA≤3 and >3 (Student's t-test + Bonferroni correction).

CpG	Student's t-test	UCSC_RefGene_Accession	UCSC_RefGene_Group	UCSC_CpG_Islands_Name
cg23475244	2.27366E-12	NA	NA	NA
cg06115835	1.05058E-11	SH3RF3	NM_001099289	Body
cg16278828	2.75829E-11	MAN2C1	NM_006715	Body
cg16639311	2.96689E-11	NA	NA	NA
cg00882175	6.86666E-11	NA	NA	NA
cg20009378	1.31144E-10	GRK1	NM_002929	Body
cg03438024	1.50385E-10	NA	NA	NA
cg11565042	1.57477E-10	GPR45	NM_007227	1stExon
cg07638362	1.67285E-10	NA	NA	NA
cg08913530	2.63749E-10	C10orf129	NM_207321	Body
cg13875763	3.558E-10	NA	NA	NA
cg02951880	4.2286E-10	VANGL2	NM_020335	Body
cg19450145	4.28193E-10	TULP4;TULP4	NM_020245;NM_001007466	Body;Body
cg06199907	4.28514E-10	MARVELD3	NM_001017967	Body
cg00472528	5.89409E-10	NA	NA	NA
cg10857489	5.98209E-10	SLC12A7	NM_006598	Body
cg23162967	6.9311E-10	CBLB	NM_170662	TSS200
cg06609415	6.95911E-10	NA	NA	NA
cg15537788	1.02976E-09	SIPA1L3	NM_015073	3'UTR
cg16276063	1.26828E-09	SOX2OT	NR_004053	Body
cg04724873	1.50316E-09	KIAA1751	NM_001080484	Body
cg21963643	2.07292E-09	CACNA1E	NM_000721	TSS1500
cg24002010	2.4842E-09	TMEM86B;SAPS1	NM_173804;NM_014931	TSS1500;3'UTR
cg09991951	2.64527E-09	NA	NA	NA
cg09984469	2.8116E-09	CYP26B1	NM_019885	Body
cg12456777	2.81964E-09	SNORD42B;RPL23A	NR_000013;NM_000984	TSS1500;1stExon
cg18062721	3.19762E-09	VGLL4;VGLL4	NM_014667;NM_001128219	Body;Body
cg09242721	3.24334E-09	TRNT1	NM_182916	TSS200
cg20376082	3.93989E-09	NA	NA	NA
cg08531746	4.18788E-09	ATP11A;ATP11A	NM_015205;NM_032189	Body;Body
cg15944459	4.21775E-09	PLA2G6;BAIAP2L2;PLA2G6	NM_001004426;NM_025045;NM_003560	3'UTR;TSS1500;3'UTR

cg19020758	4.22943E-09	NA	NA	NA
cg19967421	5.0164E-09	XCR1;XCR1	NM_005283;NM_001024644	Body;Body
cg15999833	5.06114E-09	HECW1	NM_015052	Body
cg05571558	5.21268E-09	ATP2B2;MIR885;ATP2B2	NM_001683;NR_030614;NM_001001331	Body;TSS200;Body
cg12739914	5.7632E-09	NA	NA	NA
cg14964327	5.95968E-09	NA	NA	NA
cg03227783	8.18612E-09	HLA-DPB2	NR_001435	Body
cg00638021	8.90586E-09	COL1A1	NM_000088	Body
cg02541778	9.74904E-09	NA	NA	NA
cg18391611	1.02129E-08	SBK2	NM_001101401	Body
cg00017601	1.03419E-08	PTK2B;PTK2B;PTK2B;PTK2B	NM_173176;NM_173175;NM_173174;NM_004103	Body;Body;Body;Body
cg06778713	1.04337E-08	NA	NA	NA
cg13436417	1.1216E-08	PPAPDC1A	NM_001030059	Body
cg04772948	1.12933E-08	LOC284788;LOC284788	NR_027090;NR_027089	TSS200;TSS200
cg23402821	1.13151E-08	NA	NA	NA
cg15236063	1.15044E-08	SELK	NM_021237	3'UTR
cg10126022	1.20008E-08	NA	NA	NA
cg15966151	1.26135E-08	NR0B2	NM_021969	Body
cg21654058	1.27815E-08	CCDC137	NM_199287	3'UTR
cg01223673	1.31634E-08	INSC	NM_001031853	TSS200
cg20655342	1.50387E-08	NA	NA	NA
cg19255333	1.50577E-08	PEMT;PEMT;PEMT	NM_148172;NM_148173;NM_007169	Body;Body;Body
cg11580090	1.53505E-08	CYB5R1	NM_016243	3'UTR
cg17740822	1.67943E-08	DUSP6;DUSP6	NM_001946;NM_022652	Body;Body
cg04266929	1.71097E-08	NA	NA	NA
cg04362790	1.72259E-08	NA	NA	NA
cg08540945	1.77753E-08	NA	NA	NA
cg02194046	1.78768E-08	NA	NA	NA
cg21532334	1.93139E-08	NA	NA	NA
cg01983759	1.94675E-08	MED16	NM_005481	3'UTR
cg12404679	1.96695E-08	KLHL30	NM_198582	Body
cg15401862	1.99484E-08	KCNMB4	NM_014505	3'UTR
cg17745433	2.06416E-08	OBSCN;OBSCN	NM_052843;NM_001098623	Body;Body

cg24288751	2.07316E-08	NA	NA	NA
cg19094530	2.10288E-08	NA	NA	NA
cg12183549	2.22013E-08	LOC221710;HERV-FRD	NM_001135575;NM_207582	Body;TSS200
cg22870994	2.25906E-08	NA	NA	NA
cg10400362	2.40204E-08	AJAP1;AJAP1	NM_018836;NM_001042478	Body;Body
cg03649497	2.447E-08	CACNA1G;CACNA1G;CACNA1G;CACNA1G;CACNA1G;CACNA1G;CACNA1G;CACNA1G;CACNA1G;CACNA1G;CACNA1G;CACNA1G;CACNA1G;CACNA1G;CACNA1G	NM_198384;NM_198379;NM_198380;NM_198377;NM_198385;NM_018896;NM_198396;NM_198386;NM_198376;NM_198397;NM_198388;NM_198383;NM_198382;NM_198378;NM_198387	Body;Body;Body;Body;Body;Body;Body;Body;Body;Body;Body;Body;Body;Body;Body
cg01764559	2.62922E-08	NA	NA	NA
cg04217096	2.67583E-08	KANK4	NM_181712	TSS1500
cg17367356	2.73791E-08	MAPK8IP1	NM_005456	TSS200
cg02765496	2.89732E-08	FAM122A;PIP5K1B	NM_138333;NM_003558	TSS1500;5'UTR
cg02866076	3.02165E-08	NA	NA	NA
cg16925003	3.05511E-08	PXDN	NM_012293	Body
cg19294931	3.1771E-08	TTBK1	NM_032538	Body
cg09853839	3.17816E-08	NA	NA	NA
cg18934822	3.2196E-08	SKI	NM_003036	Body
cg13441112	3.41983E-08	NA	NA	NA
cg03820205	3.44103E-08	VDAC2	NM_003375	TSS200
cg14464244	3.7604E-08	MAGI2	NM_012301	TSS200
cg09540629	3.84003E-08	KNDC1	NM_152643	Body
cg10293354	3.84713E-08	NA	NA	NA
cg11791144	3.85635E-08	NRG1	NM_013962	Body
cg01965508	3.88554E-08	PIGR	NM_002644	TSS1500
cg22341273	3.91313E-08	HEATR7A;HEATR7A;HEATR7A	NM_001099281;NM_001099280;NM_032450	Body;Body;Body
cg18911817	3.95763E-08	NKD2	NM_033120	Body
cg08145495	3.97216E-08	FUT6;FUT6	NM_001040701;NM_000150	5'UTR;5'UTR
cg20566449	4.35244E-08	RASA3	NM_007368	Body
cg21864616	4.48546E-08	NA	NA	NA
cg02793828	4.50708E-08	NA	NA	NA
cg09869950	4.52145E-08	FGGY;FGGY	NM_018291;NM_001113411	5'UTR;5'UTR
cg00931944	4.53348E-08	NA	NA	NA
cg13397282	4.57856E-08	DAB2IP;DAB2IP	NM_138709;NM_032552	Body;Body
cg04792380	4.87879E-08	NA	NA	NA

cg23924911	4.97327E-08	NA	NA	NA
cg10379653	5.18306E-08	TBC1D1;PTTG2	NM_015173;NM_006607	Body;1stExon
cg03771840	5.35627E-08	TRIM15	NM_033229	3'UTR
cg18511558	5.40603E-08	ARHGEF10	NM_014629	Body
cg00570269	5.43131E-08	C13orf33	NM_032849	Body
cg06836020	5.44411E-08	LOC440354;LOC595101	NR_002473;NR_002453	Body;Body
cg10727660	5.56421E-08	AGAP3	NM_031946	Body
cg18100830	5.64357E-08	NA	NA	NA
cg06134974	5.82743E-08	CSRNP2	NM_030809	TSS1500
cg24613691	6.24462E-08	NA	NA	NA
cg04661591	6.52969E-08	NA	NA	NA
cg21683619	6.56135E-08	LEPR;LEPR;LEPR	NM_001003679;NM_002303;NM_001003680	Body;Body;Body
cg00830435	6.6445E-08	NA	NA	NA
cg08723637	6.75922E-08	NA	NA	NA
cg24545908	6.77123E-08	NA	NA	NA
cg18701983	6.82271E-08	TNXB	NM_019105	5'UTR
cg02263813	6.94561E-08	MT1A;MT1A	NM_005946;NM_005946	1stExon;5'UTR
cg01789926	7.08109E-08	C16orf91	NM_001010878	Body
cg13116086	7.11835E-08	POM121L10P	NR_024593	TSS1500
cg10790887	7.25235E-08	INPP5A	NM_005539	Body
cg20418501	7.25336E-08	NA	NA	NA
cg03312366	7.27935E-08	NA	NA	NA
cg22964775	7.63059E-08	LPHN3	NM_015236	TSS1500
cg07433154	7.6651E-08	NA	NA	NA
cg20752695	7.94813E-08	NA	NA	NA
cg11181726	7.96382E-08	NA	NA	NA
cg21319458	8.15635E-08	BAZ2B;BAZ2B	NM_013450;NM_013450	1stExon;5'UTR
cg11024682	8.88179E-08	SREBF1;SREBF1	NM_004176;NM_001005291	Body;Body
cg14044465	9.32479E-08	NA	NA	NA
cg14563732	9.41621E-08	TSNARE1	NM_145003	5'UTR
cg17460597	1.06124E-07	NA	NA	NA
cg08438435	1.06434E-07	APBA2;APBA2	NM_001130414;NM_005503	Body;Body
cg06954660	1.09748E-07	HLA-DOA	NM_002119	Body

cg19526076	1.1855E-07	NA	NA	NA
cg10505024	1.19543E-07	KEAP1;KEAP1	NM_203500;NM_012289	Body;Body
cg18547667	1.22085E-07	NA	NA	NA
cg13108601	1.22662E-07	FRS3;PRICKLE4	NM_006653;NM_013397	TSS1500;TSS1500
cg00031105	1.26602E-07	RXRB	NM_021976	Body
cg22957121	1.41049E-07	MGAT4C	NM_013244	5'UTR
cg05086567	1.4211E-07	MPDU1;MPDU1	NM_004870;NR_024603	TSS1500;TSS1500
cg07016356	1.45162E-07	CXCR1	NM_000634	TSS1500
cg00090197	1.60117E-07	TMEM125	NM_144626	Body
cg22901726	1.68293E-07	NA	NA	NA
cg16462528	1.76319E-07	LECT1;LECT1	NM_001011705;NM_007015	Body;Body
cg01243823	1.84771E-07	NOD2	NM_022162	Body
cg00584840	1.86037E-07	NA	NA	NA
cg21473183	1.89452E-07	GNLY;GNLY	NM_006433;NM_012483	3'UTR;3'UTR
cg00310201	1.92627E-07	ZAP70;ZAP70	NM_001079;NM_207519	Body;Body
cg25020232	1.98102E-07	NA	NA	NA
cg00366594	1.99596E-07	MIR527	NR_030219	TSS1500
cg11911399	2.07671E-07	SLC25A24;SLC25A24	NM_013386;NM_013386	1stExon;5'UTR
cg01973725	2.20784E-07	RNU5E;RNU5D;ZCCHC9;ZCCHC9;ZCCHC9	NR_002754;NR_002755;NM_032280;NM_001131036;NM_001131035	Body;Body;3'UTR;3'UTR;3'UTR
cg20011604	2.37712E-07	TUBA3C	NM_006001	Body
cg20891622	2.39133E-07	WDR82;MIRLET7G	NM_025222;NR_029660	Body;TSS1500
cg04026699	2.53236E-07	NA	NA	NA
cg18784586	2.58614E-07	GPRC5A;GPRC5A	NM_003979;NM_003979	1stExon;5'UTR
cg02883247	2.59447E-07	KIRREL3;KIRREL3	NM_001161707;NM_032531	Body;Body
cg04889221	2.6298E-07	CCDC37	NM_182628	Body
cg06423822	2.66213E-07	PTPRN2;PTPRN2;PTPRN2	NM_002847;NM_130842;NM_130843	Body;Body;Body
cg05978306	2.70876E-07	MYO1C;MYO1C;MYO1C	NM_001080779;NM_033375;NM_001080950	Body;Body;Body
cg19741148	2.82975E-07	NA	NA	NA
cg03708092	2.89339E-07	NA	NA	NA
cg04903691	2.95125E-07	CXCR5;CXCR5	NM_001716;NM_032966	Body;TSS200
cg05397362	2.97918E-07	NA	NA	NA
cg02194842	3.2617E-07	NA	NA	NA

cg08370718	3.35668E-07	DTWD1;DTWD1;DTWD1;DTWD1;C15orf33	NM_020234;NM_001144955;NM_001144955;NM_020234;NM_152647	1stExon;1stExon;5'UTR;5'UTR;TSS200
cg24677472	3.37084E-07	NA	NA	NA
cg13086025	3.47324E-07	KIAA1967;KIAA1967	NM_021174;NM_199205	Body;Body
cg20168964	3.53425E-07	TMEM132D	NM_133448	Body
cg05104581	3.67876E-07	ACSF3;ACSF3;ACSF3	NR_023316;NM_001127214;NM_174917	Body;5'UTR;5'UTR
cg22636056	3.6978E-07	NA	NA	NA
cg09422450	3.73293E-07	NA	NA	NA
cg09764443	3.75675E-07	POU2F3	NM_014352	TSS1500
cg06580551	3.99214E-07	ZNF662;ZNF662;ZNF662	NM_001134656;NM_207404;NM_207404	TSS200;5'UTR;1stExon
cg21466107	4.01298E-07	GLI3	NM_000168	Body
cg17588812	4.15505E-07	KIF1A	NM_004321	Body
cg06180061	4.20369E-07	C16orf91	NM_001010878	Body
cg04737885	4.2149E-07	ADCY9	NM_001116	3'UTR
cg04559178	4.27593E-07	NA	NA	NA
cg17484877	4.42465E-07	PTPRN2;PTPRN2;PTPRN2	NM_002847;NM_130842;NM_130843	Body;Body;Body
cg06330593	4.43392E-07	KRT34	NM_021013	Body
cg02393907	4.88982E-07	ACTR3C	NM_001164458	5'UTR
cg08204369	5.44356E-07	HN1;HN1;HN1;HN1;HN1;HN1	NM_001002033;NM_001002032;NM_001002032;NM_016185;NM_001002033;NM_016185	5'UTR;5'UTR;1stExon;5'UTR;1stExon;1stExon
cg09174741	5.47928E-07	THSD1;THSD1	NM_199263;NM_018676	TSS1500;TSS1500
cg18160291	5.75061E-07	HHAT;HHAT;HHAT;HHAT;HHAT;HHAT	NM_001170580;NM_001170564;NM_001170587;NM_001170588;NM_001122834;NM_018194	TSS1500;5'UTR;TSS200;TSS200;5'UTR;TSS200
cg07395907	5.8612E-07	NA	NA	NA
cg10525720	6.41707E-07	NA	NA	NA
cg16814416	6.43182E-07	TCTEX1D4	NM_001013632	Body
cg02048674	6.44307E-07	RPL13AP5;RPL13A	NR_026712;NM_012423	Body;Body
cg00658449	6.48062E-07	BEYLA;BEYLA	NR_027012;NR_027013	TSS1500;TSS1500
cg13885205	6.52385E-07	NA	NA	NA
cg20936321	6.59944E-07	NA	NA	NA
cg09872774	6.66606E-07	NR2E3;NR2E3	NM_014249;NM_016346	Body;Body
cg12853981	6.69682E-07	TOMM20L	NM_207377	1stExon
cg13238876	6.77107E-07	NA	NA	NA
cg19385725	7.04686E-07	NPC1	NM_000271	Body
cg05063097	7.0554E-07	ZNF423	NM_015069	TSS1500

cg20186995	7.64286E-07	NA	NA	NA
cg03531754	7.67304E-07	FAM83A;FAM83A	NM_032899;NM_207006	TSS1500;TSS1500
cg00097968	7.81125E-07	RPTN	NM_001122965	3'UTR
cg08052428	7.84066E-07	RALGDS;RALGDS	NM_001042368;NM_006266	Body;1stExon
cg03705947	7.96725E-07	GADD45A	NM_001924	Body
cg17362483	8.0517E-07	NA	NA	NA
cg03871124	8.29958E-07	NA	NA	NA
cg05567435	8.34919E-07	METTL7B	NM_152637	TSS1500
cg14750501	8.53962E-07	CCNE1;CCNE1;CCNE1	NM_057182;NM_001238;NM_001238	TSS1500;5'UTR;1stExon
cg04618340	8.59408E-07	NA	NA	NA
cg13241473	8.61287E-07	TFEB;TFEB	NM_001167827;NM_007162	5'UTR;TSS1500
cg20038204	8.63952E-07	SETBP1	NM_015559	Body
cg02694576	8.64878E-07	PTDSS2	NM_030783	Body
cg04138756	8.79843E-07	SPRR3;SPRR3	NM_001097589;NM_005416	TSS1500;TSS1500
cg15305352	8.92336E-07	NA	NA	NA
cg02021157	8.92661E-07	KCNQ1;KCNQ1	NM_000218;NM_181798	Body;Body
cg02304481	9.09056E-07	FAM178B;FAM178B	NM_001122646;NM_001122646	5'UTR;1stExon
cg25214900	9.16871E-07	SYT1;SYT1;SYT1	NM_005639;NM_001135805;NM_001135806	Body;Body;Body
cg09887867	9.23434E-07	NA	NA	NA
cg12438413	9.32189E-07	RARA;RARA;RARA;RARA	NM_001145301;NM_000964;NM_001024809;NM_001145302	Body;Body;Body;Body
cg05422174	9.42446E-07	KIRREL3;KIRREL3	NM_001161707;NM_032531	Body;Body
cg06611444	9.57503E-07	NA	NA	NA
cg23104823	1.0005E-06	PRPF39;PRPF39	NM_017922;NM_017922	1stExon;5'UTR
cg06883956	1.00222E-06	MEGF6;MIR551A	NM_001409;NR_030277	Body;Body
cg08266945	1.03039E-06	C2orf77;C2orf77;PHOSPHO2;KLHL23	NM_001085447;NM_001085447;NM_001008489;NM_144711	5'UTR;1stExon;TSS200;TSS200
cg04948475	1.04455E-06	UGT2A3;UGT2A3	NM_024743;NR_024010	Body;Body
cg22753607	1.0612E-06	ZCCHC7	NM_032226	TSS200
cg17633683	1.0688E-06	SLC6A18	NM_182632	TSS200
cg17599958	1.07525E-06	AP2A2	NM_012305	Body
cg08980198	1.08186E-06	NA	NA	NA
cg13426340	1.10395E-06	NA	NA	NA
cg09015484	1.1224E-06	NA	NA	NA
cg23205818	1.15944E-06	RGS6	NM_004296	Body

cg08073153	1.16831E-06	LEMD2;LEMD2	NM_181336;NM_001143944	Body;TSS200
cg22920258	1.24438E-06	MCHR1	NM_005297	TSS1500
cg11342452	1.25461E-06	NKX6-2	NM_177400	TSS1500
cg13896328	1.28565E-06	PTPRU;PTPRU;PTPRU	NM_005704;NM_133178;NM_133177	3'UTR;3'UTR;3'UTR
cg21120176	1.30498E-06	LOC285780	NR_026970	Body
cg21367769	1.33453E-06	SCGN	NM_006998	TSS1500
cg03900195	1.34178E-06	CHAF1B	NM_005441	5'UTR
cg17767933	1.35476E-06	NA	NA	NA
cg07835232	1.37663E-06	DENND2A	NM_015689	TSS1500
cg22583147	1.45322E-06	C3orf77	NM_001145030	Body
cg19500057	1.54155E-06	STRN3;STRN3;MIR624	NM_001083893;NM_014574;NR_030354	Body;Body;TSS1500
cg22081084	1.64058E-06	LRRC8B	NM_001134476	TSS200
cg03209809	1.7336E-06	CHCHD6	NM_032343	TSS1500
cg06167524	1.77811E-06	NA	NA	NA
cg13733010	1.86153E-06	ZNF716	NM_001159279	3'UTR
cg23661013	1.88315E-06	HNF1A	NM_000545	TSS1500
cg07145255	2.01286E-06	DEPDC1B;DEPDC1B	NM_018369;NM_001145208	Body;Body
cg19418922	2.04665E-06	EXT2;EXT2	NM_000401;NM_207122	3'UTR;3'UTR
cg04238871	2.0736E-06	FAM53B	NM_014661	5'UTR
cg21151017	2.09906E-06	NA	NA	NA
cg24146685	2.28164E-06	MOGAT3	NM_178176	TSS1500
cg23991169	2.30916E-06	PCGF3	NM_006315	Body
cg00170564	2.31057E-06	BTBD11	NM_001018072	Body
cg00397871	2.39515E-06	CCDC129	NM_194300	3'UTR
cg03694795	2.43155E-06	SC65	NM_006455	Body
cg17780691	2.53023E-06	MYH3	NM_002470	Body
cg03399598	2.53306E-06	GALNT7	NM_017423	Body
cg04621069	2.59815E-06	ECE1;ECE1;ECE1;ECE1	NM_001113347;NM_001397;NM_001113348;NM_001113349	3'UTR;3'UTR;3'UTR;3'UTR
cg11057205	2.64106E-06	NA	NA	NA
cg23784907	2.65508E-06	PATL1	NM_152716	TSS200
cg05935374	2.72558E-06	RAB30	NM_014488	TSS200
cg14028671	2.72906E-06	NPDC1	NM_015392	Body
cg23789977	2.75552E-06	NA	NA	NA

cg08458400	2.76783E-06	CNR1;CNR1;CNR1;CNR1;CNR1;CNR1	NM_001160259;NM_001160226;NM_001160258;NM_001160260;NM_033181;NM_016083	Body;Body;Body;Body;Body;Body
cg01841050	2.9369E-06	KRT34	NM_021013	TSS1500
cg13554030	2.96963E-06	LSM2	NM_021177	Body
cg21457856	3.01714E-06	NA	NA	NA
cg12484590	3.01996E-06	NA	NA	NA
cg16198129	3.14845E-06	ZNF662;ZNF662	NM_207404;NM_001134656	Body;Body
cg15877295	3.20094E-06	UBN2	NM_173569	TSS1500
cg00832650	3.30614E-06	FLNC;FLNC	NM_001127487;NM_001458	Body;Body
cg20451986	3.44104E-06	NA	NA	NA
cg17126142	3.49708E-06	NA	NA	NA
cg09198085	3.76421E-06	AK7	NM_152327	Body
cg21950037	3.8386E-06	NA	NA	NA
cg14156446	3.99672E-06	NR6A1;NR6A1	NM_033334;NM_001489	3'UTR;3'UTR
cg02051981	4.00597E-06	PAPPA2;PAPPA2	NM_021936;NM_020318	TSS200;TSS200
cg17361449	4.01746E-06	TTPAL;TTPAL	NM_001039199;NM_024331	TSS200;TSS200
cg07036918	4.06634E-06	SCUBE2;SCUBE2	NM_001170690;NM_020974	TSS200;TSS200
cg21304211	4.08443E-06	C1orf183;C1orf183;C1orf183	NM_198926;NM_019099;NM_019099	Body;1stExon;5'UTR
cg19932227	4.13353E-06	ADPRH	NM_001125	TSS200
cg24059022	4.25327E-06	SLC12A7	NM_006598	Body
cg12884982	4.42689E-06	JPH1	NM_020647	Body
cg07797833	4.56158E-06	NA	NA	NA
cg19391498	4.62377E-06	GALM	NM_138801	TSS1500
cg15603885	4.76007E-06	ABRA	NM_139166	1stExon
cg12616745	4.77849E-06	MFSD6	NM_017694	TSS200
cg20703375	4.85353E-06	ARPC1A	NM_006409	Body
cg01335566	5.20462E-06	CHD5	NM_015557	Body
cg24420395	5.22221E-06	DUSP4;DUSP4	NM_001394;NM_057158	Body;TSS1500
cg22129122	5.22745E-06	NA	NA	NA
cg13116438	5.27667E-06	STK19;STK19;DOM3Z;STK19	NM_004197;NM_032454;NM_005510;NR_026717	Body;Body;TSS1500;Body
cg01809437	5.55608E-06	KLHL36	NM_024731	Body
cg13695383	5.64626E-06	AGFG2	NM_006076	Body
cg12610079	5.67609E-06	C1orf110	NM_178550	TSS1500

cg10508347	5.73951E-06	SLC45A1	NM_001080397	1stExon
cg24511898	5.80904E-06	ATG4B;ATG4B;THAP4	NM_178326;NM_013325;NM_015963	TSS1500;TSS1500;TSS200
cg23061412	5.87491E-06	MIR518A2	NR_030213	Body
cg00232805	5.91099E-06	ANKRD36	NM_001164315	1stExon
cg07312954	6.05547E-06	DENND4B	NM_014856	Body
cg09468777	6.18052E-06	PDE6B;PDE6B;PDE6B	NM_000283;NM_001145291;NM_001145292	Body;Body;Body
cg17746791	6.23926E-06	PIM1	NM_002648	TSS200
cg13412395	6.28914E-06	NR4A3;NR4A3;NR4A3	NM_006981;NM_173198;NM_173199	TSS1500;TSS1500;TSS1500
cg19613905	6.30348E-06	MNS1;MNS1	NM_018365;NM_018365	1stExon;5'UTR
cg10901867	6.36201E-06	NAT8L	NM_178557	Body
cg10150615	6.3934E-06	LOC391322	NM_001144931	TSS200
cg11973777	6.54805E-06	NA	NA	NA
cg20087917	6.67372E-06	C14orf133;AHSA1	NM_022067;NM_012111	TSS200;TSS1500
cg02180424	6.7242E-06	MDGA2	NM_001113498	Body
cg13135459	6.77115E-06	LOC387646	NR_003525	TSS200
cg11332951	6.8941E-06	NA	NA	NA
cg13611636	7.06685E-06	ZMAT4;ZMAT4	NM_001135731;NM_024645	5'UTR;5'UTR
cg16476048	7.11874E-06	C10orf122	NM_001128202	TSS1500
cg08862564	7.22418E-06	CREB1;CREB1	NM_134442;NM_004379	5'UTR;5'UTR
cg09864156	7.3205E-06	SUPV3L1	NM_003171	TSS1500
cg25188165	7.61478E-06	NA	NA	NA
cg07982899	7.64326E-06	C4orf22	NM_152770	Body
cg13713052	7.81475E-06	FAM162A;CCDC58	NM_014367;NM_001017928	TSS1500;TSS200
cg09529537	8.10782E-06	JMY	NM_152405	TSS200
cg09379601	8.10817E-06	DNASE2;DNASE2	NM_001375;NM_001375	5'UTR;1stExon
cg02021544	8.13159E-06	MYLK3	NM_182493	TSS1500
cg10187692	8.43411E-06	VPS13B;VPS13B	NM_017890;NM_152564	Body;Body
cg24162465	8.47196E-06	NA	NA	NA
cg21689228	8.59085E-06	MSX1	NM_002448	TSS1500
cg00402074	8.77524E-06	GPR116;GPR116	NM_001098518;NM_015234	TSS200;5'UTR
cg13507084	8.77606E-06	CNTFR;CNTFR	NM_147164;NM_001842	Body;Body
cg05626927	9.23777E-06	NA	NA	NA
cg23851043	9.30095E-06	MOBKL3;MOBKL3;MOBKL3	NM_199482;NM_001100819;NM_015387	5'UTR;Body;Body

cg02133350	9.37879E-06	PMS2;PMS2	NM_000535;NR_003085	Body;Body
cg13944397	9.3883E-06	NA	NA	NA
cg04324509	9.44288E-06	PCNP	NM_020357	Body
cg08604596	9.53618E-06	CCDC101	NM_138414	5'UTR
cg18181431	9.81108E-06	NA	NA	NA
cg13265914	9.81734E-06	REEP4	NM_025232	TSS200
cg18420708	9.91403E-06	EPHX3;EPHX3	NM_024794;NM_001142886	Body;Body
cg05744073	9.98333E-06	MIR132	NR_029674	Body
cg05254095	1.01477E-05	ZDHHC7;ZDHHC7	NM_001145548;NM_017740	3'UTR;3'UTR
cg07306531	1.02802E-05	ZNF75A	NM_153028	TSS200
cg07908574	1.05373E-05	SLC45A1	NM_001080397	Body
cg21273856	1.09993E-05	NCOA7;NCOA7;NCOA7;NCOA7	NM_181782;NM_001122842;NM_181782;NM_001122842	5'UTR;5'UTR;1stExon;1stExon
cg10082357	1.14906E-05	ATP6V1F	NM_004231	3'UTR
cg23003225	1.16586E-05	NA	NA	NA
cg07723459	1.17152E-05	NA	NA	NA
cg09311052	1.19085E-05	UBE2Q1	NM_017582	TSS1500
cg23330006	1.19095E-05	NUAK1	NM_014840	Body
cg17461336	1.19316E-05	CYP3A43;CYP3A43;CYP3A43	NM_022820;NM_057096;NM_057095	Body;Body;Body
cg12315590	1.19573E-05	C3orf37;C3orf37	NM_001006109;NM_020187	TSS1500;TSS200
cg11814422	1.20162E-05	SNORD58A;RPL17;SNORD58B;RPL17	NR_002571;NM_000985;NR_002572;NM_001035006	TSS1500;5'UTR;TSS200;5'UTR
cg20217938	1.24163E-05	NA	NA	NA
cg15084903	1.24465E-05	THEG;THEG	NM_016585;NM_199202	TSS200;TSS200
cg23413289	1.24614E-05	FAT1	NM_005245	Body
cg07650554	1.25627E-05	SEPHS2	NM_012248	TSS200
cg18219432	1.29432E-05	PTPRN2;PTPRN2;PTPRN2	NM_002847;NM_130842;NM_130843	Body;Body;Body
cg16692534	1.2954E-05	FOXK2	NM_004514	Body
cg21607453	1.3026E-05	NCRNA00092	NR_024129	Body
cg02956660	1.30293E-05	HSPG2	NM_005529	Body
cg22136672	1.31467E-05	NA	NA	NA
cg09016822	1.3212E-05	IER3	NM_003897	TSS200
cg03045325	1.33837E-05	MACROD1;FLRT1	NM_014067;NM_013280	Body;TSS200
cg16020249	1.36047E-05	AHDC1	NM_001029882	TSS1500
cg19545348	1.39223E-05	RCOR3;RCOR3;RCOR3;RCOR3	NM_018254;NM_001136224;NM_001136225;NM_001136223	Body;Body;Body;Body

cg08015507	1.40975E-05	NCKIPSD;NCKIPSD	NM_016453;NM_184231	Body;Body
cg05947499	1.44044E-05	TPCN1;TPCN1	NM_001143819;NM_017901	Body;Body
cg14039479	1.44161E-05	GPR26	NM_153442	3'UTR
cg09392827	1.44505E-05	NA	NA	NA
cg17321888	1.45694E-05	NA	NA	NA
cg00455514	1.46261E-05	NA	NA	NA
cg08571347	1.52188E-05	NA	NA	NA
cg02902865	1.5242E-05	DHRS3	NM_004753	TSS1500
cg01353569	1.53128E-05	HBP1	NM_012257	5'UTR
cg16925090	1.55973E-05	KIAA1377;ANGPTL5	NM_020802;NM_178127	TSS1500;5'UTR
cg05318427	1.56639E-05	KCMF1	NM_020122	Body
cg08664652	1.58441E-05	GOSR1;GOSR1;GOSR1	NM_001007024;NM_004871;NM_001007025	TSS200;TSS200;TSS200
cg00459550	1.5942E-05	NA	NA	NA
cg18006626	1.61122E-05	NA	NA	NA
cg16300033	1.6231E-05	ANKRD30A	NM_052997	TSS1500
cg16542646	1.65887E-05	NA	NA	NA
cg14054990	1.66351E-05	KRTAP19-5	NM_181611	TSS200
cg06451949	1.67147E-05	NA	NA	NA
cg03157738	1.73712E-05	KANK2	NM_001136191	5'UTR
cg05639679	1.82168E-05	KIAA1522	NM_020888	Body
cg09564133	1.83058E-05	MXD3;MXD3;MXD3;MXD3	NM_031300;NM_001142935;NM_031300;NM_001142935	1stExon;1stExon;5'UTR;5'UTR
cg25256723	1.84336E-05	F5	NM_000130	TSS200
cg16104446	1.86036E-05	MCM4;MCM4;PRKDC;PRKDC	NM_182746;NM_005914;NM_001081640;NM_006904	Body;Body;TSS1500;TSS1500
cg01334186	1.86861E-05	PSMB4	NM_002796	Body
cg17204834	1.89725E-05	ZNF589	NM_016089	TSS200
cg19455335	1.90926E-05	C8orf58	NM_001013842	Body
cg15649111	1.94177E-05	CPA5;CPA5;CPA5	NM_001127441;NM_001127442;NM_080385	Body;Body;Body
cg11688874	1.94417E-05	WAC;WAC;WAC;WAC;WAC	NM_100486;NM_016628;NM_016628;NM_100486;NR_024557	1stExon;5'UTR;1stExon;5'UTR;Body
cg17586419	1.95313E-05	TOP1P2;PIWIL3	NR_001283;NM_001008496	TSS1500;5'UTR
cg21687563	1.97818E-05	TTC28	NM_001145418	Body
cg11208039	2.02989E-05	ZDHHC13;ZDHHC13;ZDHHC13;ZDHHC13	NM_001001483;NM_019028;NM_001001483;NM_019028	1stExon;5'UTR;5'UTR;1stExon
cg14253896	2.08362E-05	NA	NA	NA
cg18350497	2.10935E-05	C11orf54	NM_014039	5'UTR

cg08788291	2.16706E-05	RECQL5;LOC643008	NM_004259;NM_001162995	Body;TSS1500
cg13749939	2.27373E-05	SLC25A22	NM_024698	TSS1500
cg04517374	2.32798E-05	CABLES2	NM_031215	Body
cg11530914	2.41973E-05	FHOD1;SLC9A5	NM_013241;NM_004594	TSS200;TSS1500
cg06900676	2.4586E-05	KIAA1841;KIAA1841	NM_001129993;NM_032506	5'UTR;TSS200
cg02581828	2.46746E-05	NA	NA	NA
cg19240857	2.47576E-05	LY6G6E;LY6G6E;LY6G6D	NR_003673;NR_024541;NM_021246	TSS1500;TSS1500;TSS200
cg01093065	2.48121E-05	PLEKHA4;PLEKHA4	NM_001161354;NM_020904	TSS1500;TSS1500
cg21104686	2.55396E-05	NA	NA	NA
cg15244101	2.64908E-05	NA	NA	NA
cg12402495	2.79807E-05	C1orf43;C1orf43;UBAP2L;UBAP2L;C1orf43	NM_015449;NM_138740;NM_014847;NM_001127320;NM_001098616	1stExon;1stExon;TSS1500;5'UTR;1st Exon
cg12639422	2.80525E-05	UGP2;UGP2;UGP2	NM_006759;NM_006759;NM_001001521	1stExon;5'UTR;5'UTR
cg18624108	2.84891E-05	COL11A2;COL11A2;COL11A2	NM_080679;NM_080681;NM_080680	Body;Body;Body
cg01903185	2.85556E-05	FGFR3;FGFR3;FGFR3	NM_001163213;NM_000142;NM_022965	Body;Body;Body
cg05608508	2.90887E-05	NTAN1	NM_173474	Body
cg03797768	2.91474E-05	NA	NA	NA
cg14840863	2.98555E-05	COLEC11;COLEC11	NM_024027;NM_199235	Body;Body
cg14842398	3.0767E-05	KIAA0182	NM_001134473	5'UTR
cg24969222	3.09249E-05	NA	NA	NA
cg13789711	3.16619E-05	PHPT1;PHPT1	NM_001135861;NM_014172	TSS200;TSS200
cg08470053	3.30327E-05	ZNF160;ZNF160;ZNF160	NM_033288;NM_198893;NM_001102603	5'UTR;5'UTR;5'UTR
cg19597318	3.30698E-05	NFASC;NFASC;NFASC;NFASC;NFASC	NM_015090;NM_001160333;NM_001005388;NM_001005389;NM_001160332	5'UTR;5'UTR;5'UTR;5'UTR;5'UTR
cg01078248	3.31679E-05	NDUFA5	NM_005000	Body
cg09391949	3.32352E-05	JPH3	NM_020655	3'UTR
cg01374398	3.32658E-05	SCAP	NM_012235	5'UTR
cg02895995	3.37781E-05	PEX11G	NM_080662	TSS200
cg19260567	3.38472E-05	CNTNAP2	NM_014141	Body
cg06788172	3.38837E-05	DMXL2	NM_015263	TSS200
cg19881032	3.39593E-05	SLC16A8	NM_013356	TSS200
cg20248822	3.55477E-05	C1orf151	NM_001032363	TSS1500
cg14476101	3.58678E-05	PHGDH	NM_006623	Body
cg16139199	3.62933E-05	ESCO1	NM_052911	5'UTR

cg21515243	3.6516E-05	RIOK3;RIOK3	NM_003831;NM_003831	1stExon;5'UTR
cg14457132	3.65832E-05	CAGE1;CAGE1;CAGE1;CAGE1;CAGE1;CAGE1;RIOK1	NM_001170693;NM_001170693;NM_001170692;NM_205864;NM_001170692;NM_205864;NM_031480	1stExon;5'UTR;5'UTR;1stExon;1stExon;5'UTR;TSS200
cg19407545	3.68515E-05	NA	NA	NA
cg05072951	3.68693E-05	CAPN1;CAPN1	NM_005186;NM_005186	1stExon;5'UTR
cg09981884	3.72045E-05	TMEM129;TMEM129	NM_138385;NM_001127266	Body;Body
cg23182539	3.8011E-05	TIAL1;TIAL1	NM_001033925;NM_003252	Body;Body
cg02841941	3.83937E-05	P2RY1	NM_002563	TSS200
cg20412283	3.87444E-05	DDAH2	NM_013974	5'UTR
cg23319790	4.13426E-05	MIR519D;MIR517A	NR_030202;NR_030201	TSS1500;TSS200
cg00334821	4.13649E-05	LIMK1	NM_002314	Body
cg07022307	4.27829E-05	ATP1A4;ATP1A4	NM_001001734;NM_144699	TSS1500;Body
cg03609216	4.30037E-05	TRIM15	NM_033229	1stExon
cg08846459	4.30904E-05	NA	NA	NA
cg02582774	4.36382E-05	COQ5	NM_032314	Body
cg04598128	4.47278E-05	NA	NA	NA
cg00986762	4.6156E-05	PSMB2;PSMB2	NM_002794;NM_002794	1stExon;5'UTR
cg16209873	4.70209E-05	OR52W1	NM_001005178	TSS1500
cg13442432	4.71524E-05	NA	NA	NA
cg12937438	4.71771E-05	NA	NA	NA
cg24250234	4.73595E-05	NA	NA	NA
cg09878914	4.81037E-05	BAT1;BAT1	NM_080598;NM_004640	Body;Body
cg09896298	4.82183E-05	DUPD1	NM_001003892	TSS200
cg06665941	4.82581E-05	IFNAR2;IFNAR2;IFNAR2	NM_207585;NM_207584;NM_000874	5'UTR;5'UTR;5'UTR
cg05353415	4.83533E-05	GLI3	NM_000168	5'UTR
cg18543035	4.98898E-05	NA	NA	NA
cg02881186	5.10707E-05	TRAPPC9;TRAPPC9	NM_001160372;NM_031466	Body;Body
cg20261243	5.12876E-05	NCOA5	NM_020967	TSS200
cg06734816	5.12966E-05	NT5DC1	NM_152729	Body
cg01206944	5.24292E-05	ATP10A	NM_024490	Body
cg01258793	5.24975E-05	MAD1L1;MAD1L1;MAD1L1	NM_003550;NM_001013837;NM_001013836	Body;Body;Body
cg11596580	5.25558E-05	PRMT8;PRMT8	NM_019854;NM_019854	1stExon;5'UTR
cg18342900	5.28019E-05	C16orf87	NM_001001436	1stExon

cg19819135	5.28108E-05	SFRS11	NM_004768	Body
cg06963308	5.39577E-05	NA	NA	NA
cg14007221	5.45473E-05	FRMPD1	NM_014907	5'UTR
cg14138312	5.48704E-05	KDM4C;KDM4C;KDM4C;KDM4C	NM_015061;NM_001146694;NM_001146695;NM_001146696	Body;Body;Body;Body
cg20303815	5.50609E-05	NA	NA	NA
cg16815882	5.54133E-05	KIAA0319L	NM_024874	Body
cg25233569	5.56696E-05	RHOBTB1;RHOBTB1;RHOBTB1;RHOBTB1;RHOBTB1	NR_024555;NM_014836;NM_014836;NR_024554;NR_024556	Body;1stExon;5'UTR;Body;Body
cg19775406	5.60252E-05	MIS12;MIS12;DERL2	NM_024039;NM_024039;NM_016041	5'UTR;1stExon;TSS1500
cg18669135	5.62547E-05	NA	NA	NA
cg18181703	5.6629E-05	SOCS3	NM_003955	Body
cg02668984	5.74385E-05	PDK2	NM_002611	TSS1500
cg03160445	5.74437E-05	LNP1;TOMM70A;LNP1	NM_001085451;NM_014820;NM_001085451	5'UTR;TSS200;1stExon
cg01141459	5.7446E-05	HORMAD2;HORMAD2	NM_152510;NM_152510	1stExon;5'UTR
cg00004939	5.78357E-05	C14orf181;C14orf181	NM_207442;NM_207442	3'UTR;1stExon
cg04428700	5.83483E-05	HOXA13	NM_000522	1stExon
cg21939482	5.84953E-05	C7orf70	NM_001037163	5'UTR
cg16053745	5.87734E-05	C3orf43	NM_001077657	Body
cg01489057	5.91874E-05	DMBT1;DMBT1;DMBT1	NM_007329;NM_004406;NM_017579	Body;Body;Body
cg03811519	5.92466E-05	NUP214	NM_005085	Body
cg20980271	5.9684E-05	CLCC1;CLCC1	NM_001048210;NM_015127	TSS1500;TSS1500
cg11755407	6.09251E-05	EML3;MTA2	NM_153265;NM_004739	Body;TSS1500
cg22312050	6.141E-05	NA	NA	NA
cg10251347	6.23879E-05	NRD1;NRD1;NRD1;NRD1	NM_002525;NM_002525;NM_001101662;NM_001101662	5'UTR;1stExon;1stExon;5'UTR
cg21850852	6.25084E-05	ITGB4;ITGB4;ITGB4	NM_001005731;NM_001005619;NM_000213	Body;Body;Body
